# A metagenome-wide study of the gut virome in chronic kidney disease

**DOI:** 10.7150/thno.101601

**Published:** 2025-01-02

**Authors:** Pan Zhang, Ruochun Guo, Shiyang Ma, Hongli Jiang, Qiulong Yan, Shenghui Li, Kairuo Wang, Jiang Deng, Yanli Zhang, Yue Zhang, Guangyang Wang, Lei Chen, Lu Li, Xiaoyan Guo, Gang Zhao, Longbao Yang, Yan Wang, Jian Kang, Shanshan Sha, Shao Fan, Lin Cheng, Jinxin Meng, Hailong Yu, Fenrong Chen, Danni He, Jinhai Wang, Shuxin Liu, Haitao Shi

**Affiliations:** 1 Department of Gastroenterology, The Second Affiliated Hospital of Xi'an Jiaotong University; Shaanxi Key Laboratory of Gastrointestinal Motility Disorders; Shaanxi Provincial Clinical Research Center for Gastrointestinal Diseases; Digestive Disease Quality Control Center of Shaanxi Province, Xi'an 710004, China.; 2 College of Basic Medical Sciences, Dalian Medical University, Dalian 116044, China.; 3 Puensum Genetech Institute, Wuhan 430076, China.; 4 Department of Nephrology, Dalian Municipal Central Hospital affiliated with Dalian University of Technology, Dalian Key Laboratory of Intelligent Blood Purification, Dalian 116033, China.; 5 Department of Critical Care Nephrology and Blood Purification, the First Affiliated Hospital of Xi'an Jiaotong University, Shaanxi, 710061, China.; 6 Department of Urology, Affiliated Zhongshan Hospital of Dalian University, Dalian 116001, China.

**Keywords:** gut virome, viral operational taxonomic units, deep metagenomic sequencing, chronic kidney disease, viral function

## Abstract

**Rationale:** Chronic kidney disease (CKD) is a progressively debilitating condition leading to kidney dysfunction and severe complications. While dysbiosis of the gut bacteriome has been linked to CKD, the alteration in the gut viral community and its role in CKD remain poorly understood.

**Methods:** Here, we characterize the gut virome in CKD using metagenome-wide analyses of faecal samples from 425 patients and 290 healthy individuals.

**Results:** CKD is associated with a remarkable shift in the gut viral profile that occurs regardless of host properties, disease stage, and underlying diseases. We identify 4,649 differentially abundant viral operational taxonomic units (vOTUs) and reveal that some CKD-enriched viruses are closely related to gut bacterial taxa such as *Bacteroides*, *[Ruminococcus]*, *Erysipelatoclostridium*, and* Enterocloster* spp. In contrast, CKD-depleted viruses include more *crAss-like* viruses and often target *Faecalibacterium*, *Ruminococcus*, and *Prevotella* species. Functional annotation of the vOTUs reveals numerous viral functional signatures associated with CKD, notably a marked reduction in nicotinamide adenine dinucleotide (NAD^+^) synthesis capacity within the CKD-associated virome. Furthermore, most CKD viral signatures are reproducible in the gut viromes of diabetic kidney disease and several other common diseases, highlighting the considerable universality of disease-associated viromes.

**Conclusions:** This research provides comprehensive resources and novel insights into the CKD-associated gut virome, offering valuable guidance for future mechanistic and therapeutic investigations.

## Introduction

Chronic kidney disease (CKD) is defined as a decrease in kidney function (reduced glomerular filtration rate) or kidney damage lasting at least three months 1. This condition has already created a major public health burden, affecting approximately 10% of adults worldwide and resulting in 1.2 million deaths annually 2. By 2040, CKD is projected to be the fifth leading cause of death globally 3. Numerous risk factors contribute to CKD, including diabetes, hypertension, glomerulonephritis, cystic kidney disease and inappropriate medication use 4-7. As a progressive disease, CKD increases all-cause mortality and often leads to systemic complications, such as cardiovascular disease, mineral bone disorder, arterial hypertension, and anaemia 6, 8, and can progress to end-stage renal disease (ESRD) requiring dialysis or renal replacement therapy.

The aetiology of CKD remains largely unclear, likely influenced by both genetic and environmental factors 9-11. The gut microbiota, reflecting environmental influences, plays a critical role in the pathogenesis and progression of kidney diseases. Metabolites produced by gut microbes can regulate host physiology and kidney functions 12. Additionally, immune system components (e.g., lymphocytes, monocytes, and cytokines) facilitate communication between the gut and the kidney 13, 14. These interactions, termed the “gut-kidney axis” 15, 16, act as an essential regulator in maintaining the host's metabolic and immunological balance. Direct links between CKD and the gut microbiota have been established through high-throughput sequencing of faecal samples, revealing that CKD patients have an altered gut microbiota characterized by an increase in harmful bacteria and a decrease in probiotics 17-19. In ESRD patients, the gut microbiota shows significant aberrations, promoting the accumulation of uraemic toxins that worsen disease progression 20. Recent research has also highlighted the connection between the gut mycobiome, faecal metabolome, and serum metabolome in ESRD patients 21.

Despite the established association between gut microbiota and CKD, the composition and function of the gut viral community in CKD patients remain poorly understood. Viruses exhibit vast diversity within the human intestine. A recent study, using the faecal metagenome datasets of approximately 2,000 individuals, identified over 33,000 species-level viral populations, showcasing the extensive breadth of the human gut viral community 22. Concurrently, large-scale exploration of publicly available faecal metagenomes reconstructed over 140,000 unique viral genomes, revealing 280 viral clades prevalent worldwide 23. While viral genomes are typically small, they represent an important reservoir of genetic diversity in the ecosystem, facilitating lateral gene transfer of virulence factors, antibiotic resistance, and metabolic traits among microorganisms (e.g., bacteria and archaea) 24. Some viruses are thought to exert immunomodulatory effects due to their intrinsic anti-inflammatory properties and ability to adhere to mucosal surfaces, allowing translocation to various tissues 25, 26. The overall profile of the gut viral community (referred to as the gut virome) is relatively stable but can rapidly change in response to shifts in the host's physical state or environment 27, 28. Consequently, the dynamics of the gut virome correlate closely with various diseases, including colorectal cancer (CRC) 29, 30, inflammatory bowel disease (IBD) 31-33, liver diseases 34, 35, and autoimmune diseases 36-38. Additionally, viruses encode unique auxiliary metabolic genes (AMGs) that may influence the metabolic and immunomodulatory capabilities of the microbiota, potentially affecting the risk of developing rheumatoid arthritis (RA) 39. These findings suggest connections between the gut virome and kidney function, highlighting the need to evaluate the pathophysiological role of the gut virome in CKD patients and the gut-kidney axis.

In this study, we devised and undertook a metagenome-wide exploration of the gut virome in CKD based on faecal metagenomic datasets from 425 patients and 290 healthy controls (HCs). We utilized these data to create a study-specific viral catalogue, pinpointed numerous viral and functional signatures associated with CKD, and expanded our findings to encompass CKD-associated viral signatures in diverse common diseases. Our results offer a comprehensive view of the CKD gut virome and provide a paradigm for future studies on the virome in other relevant disorders.

## Materials and Methods

### Subjects and data processing

The methods for subject recruitment, specimen collection, faecal DNA extraction, and whole-metagenome shotgun sequencing have been detailed in previous studies 19, 20. Raw metagenomic datasets were downloaded from the National Center for Biotechnology Information (NCBI) Sequence Read Archive (SRA) under project accession IDs PRJNA449784 and PRJEB65297 for Beijing and Shanghai cohorts, respectively. The Beijing cohort comprised 254 haemodialytic CKD patients and 179 healthy controls, while the Shanghai cohort included 111 healthy volunteers and 171 patients diagnosed as CKD stage 3 (n = 12), CKD stage 4 (n = 4), non-dialyzed CKD stage 5 (n = 31), and haemodialytic CKD (n = 124). In both cohorts, demographic characteristics such as sex, age, body mass index (BMI), and dietary habits were matched between patients and controls 19, 20. CKD patients were classified based on the following criteria: stage 3, estimated glomerular filtration rate (eGFR) < 60 mL/min/1.73m^2^; stage 4, eGFR < 30 mL/min/1.73m^2^; and stage 5, eGFR < 15 mL/min/1.73m^2^. Individuals in the healthy group exhibited normal clinical parameters from routine tests (e.g., blood, urine, liver function, and renal function) and were excluded due to diseases such as hypertension, atherosclerosis, diabetes, obesity, IBD, cancer, and abnormal liver or kidney function.

Quality filtering of the raw metagenomic sequencing reads was performed using fastp v0.20.1 40 with the parameters “-q 20 -u 30 -l 90 -y --trim_poly_g”. Reads that mapped to the human genome reference (GRCh38) were removed using Bowtie2 v2.4.1 41 to eliminate human contamination. Paired-end clean reads were then assembled using MEGAHIT v1.2.9 42 with a wide range of k-mer sizes “--k-list 21,41,61,81,101,121,141”. Assembled contigs with lengths less than 5kb were discarded, while the remaining contigs were used for the identification of viral sequences.

### Identification and processing of viral sequences

The workflow of viral identification is shown in Figure S1A. Metagenomically assembled contigs were recognized as viral sequences based on their sequence features and homology to known viral genomes. Raw viral contigs were identified when they satisfied one of the following criteria: 1) identified as a virus in VIBRANT v1.2.1 43 with default parameters (-meta mode); 2) containing a greater number of viral genes than microbial genes based on searches against the CheckV marker gene set 44; or 3) achieving a score >0.9 and *P* <0.01 in DeepVirFinder v1.0 45. Contigs that were recognized as “undetermined” sequences by CheckV were discarded. Parallelly, raw provirus sequences were extracted from the contigs by CheckV, with those shorter than 5kb removed. These procedures generated a total of 178,097 candidate viral sequences (132,172 viral contigs and 45,925 proviruses). To decontaminate the viral sequences, according to the previous study 22, 46, we searched the bacterial universal single-copy orthologs (BUSCO) 47 within the raw viral sequence using hmmsearch 48 with default options and calculated the BUSCO ratio as the number of BUSCOs relevant to the total number of genes in each viral sequence. High-contaminated viral sequences with a BUSCO ratio ≥5% were removed, resulting in 170,759 sequences considered as the final viral sequences from the metagenomic samples.

The viral sequences were de-replicated based on the following steps: 1) all viral sequences were pairwise aligned using BLASTn v2.9.0 with the options “-evalue 1e-10 -word_size 20 -num_alignments 99999”; 2) viral sequences which shared 95% nucleotide identity across 75% of their length were clustered into a viral operational taxonomic unit (vOTU) using custom scripts (https://github.com/RChGO/virusDectect); 3) The longest viral sequence was considered as the representative sequence for each vOTU. Additionally, shared vOTUs between different gut virus collections (i.e., Gut Virome Database 22 and Gut Phage Database 23) were identified following the same steps as above, and the combined nonredundant vOTU catalogue (n = 45,849) was generated accordingly.

### Viral taxonomic classification and functional annotation

Viral proteins of vOTUs were predicted using Prodigal v2.6.3 49. We compiled a reference database by aggregating protein sequences from three viral databases: the Virus-Host DB 50 (downloaded in May 2021), *crAss-like* phage proteins from Guerin's study 51, and the protein catalogue from Benler's study 52. For accurate family-level taxonomic classification of viruses, we aligned the proteins of all known viral sequences from the NCBI-RefSeq database against the combined reference database using DIAMOND 53 with the options “--query-cover 50 --subject-cover 50 --id 30 --min-score 50 --max-target-seqs 10”. A viral sequence was annotated to a viral family-level taxon when over 25% of its proteins matched that family. This approach obtained an accuracy of 98.6% for family-level classification of the viruses from the NCBI-RefSeq database, which we applied to the taxonomic classification of the vOTUs. For functional analysis of viral populations, we performed BLAST searches of protein sequences of all vOTUs against the KEGG (Kyoto Encyclopedia of Genes and Genomes) database (downloaded in December 2020) using DIAMOND with the following options “--query-cover 50 --subject-cover 50 -e 1e-5 --min-score 50 --max-target-seqs 50”. The matched protein was annotated to a KEGG orthologue (KO) based on the best-hit protein.

### Virus-host prediction

In our previous work, we assembled over 19,000 high-completeness microbial genomes, representing 1,303 bacterial or archaeal species, from the 715 faecal metagenomic samples in this study 54. Based on these prokaryotic genomes, we used two approaches to implement virus-host prediction of vOTUs: the CRISPR-based approach and the homolog-based approach. For the CRISPR-based approach, firstly, the CRISPR spacer sequences of prokaryotic genomes were predicted via MinCED v0.4.2 55 with the option “-minNR 2”. All vOTUs were then aligned against the predicted CRISPR spacer sequences using BLASTn with the parameters “-evalue 1e-5 -word_size 8 -num_alignments 99999”. We retained only matches with bit-score ≥45 across the entire length of the putative CRISPR spacer sequences, assigning one or more hosts to each vOTU based on these alignment results. For the homolog-based approach, we performed alignments between vOTU sequences and host genomes using BLASTn with the options “-evalue 1e-2 -num_alignments 99999”. If the match met the criteria of ≥90% nucleotide identity over 30% coverage of vOTU, the prokaryote associated with this genome was considered as the host infected by the corresponding vOTU.

### Reads mapping rate and metagenomic profiling

We determined the read count for each vOTU in each sample by mapping clean reads to all vOTU sequences using Bowtie2 with the options “--end-to-end --fast --no-unal --no-sq --no-head”. The mapping rate of each vOTU was calculated as the read count for that vOTU divided by the total amount of clean reads in the corresponding sample. The mapping rate for each family was derived by summing the mapping rates of all vOTUs classified within that family-level taxonomy. For metagenomic profiling, to improve comparisons among samples with vastly different read counts, we randomly subsampled 2,000,000 mapped reads per sample to recalculate the read count for each vOTU. The relative abundance of each vOTU in every sample was defined as its read count divided by 2,000,000. For family-level profiles, the relative abundances of vOTUs sharing the same family-level taxonomy were added together to form the overall abundance for the family.

### Statistical analysis and data visualization

*Evaluation of the viral richness and evenness.* We calculated three diversity indexes to assess the richness and evenness of vOTUs composition in each sample. The number of observed vOTUs was defined as the count of unique vOTUs in each sample. Shannon's and Simpson's diversity indexes were calculated using the *vegan* package (function *diversity*) in the R platform. The significant difference level in diversity indexes between the two groups was analyzed using the function *wilcox.test*.

*Principal coordinates analysis (PCoA).* PCoA was performed with the R *ape* package by the function *pcoa*, and was visualized with the *ggplot2* package. To quantify similarities or dissimilarities among individuals, we generated a Bray-Curtis dissimilarity matrix (calculated by the function *vegdist* in the R *vegan* package) based on the relative abundance profiles of vOTUs.

*The impact of host factors on vOTUs composition.* The influence of host factor on vOTUs composition was assessed using permutational multivariate analysis of variance (PERMANOVA) via the function *adonis* (*vegan* package) with default arguments. PERMANOVA p-values were generated based on 1,000 permutations. In addition, we evaluated the impact of CKD status on vOTUs composition after controlling for gender, age and BMI using the function *adonis* with the argument “formula = composition ~ gender + age + BMI + disease_status”. All R-squares obtained from PERMANOVA were further adjusted by the function *RsquareAdj*.

*Identification of CKD-associated vOTUs.* To increase the reliability of the identified CKD-associated vOTUs, differential abundance analyses were performed based on two independent cohorts (Shanghai and Beijing). For each cohort, the mean relative abundances of vOTUs were used to calculate fold changes between healthy controls and CKD patients. Statistical significance was assessed using Wilcoxon rank-sum test for p-value calculation, with false discovery rate (FDR) correction applied via the function *fdrtool* in R platform 4.0.3. We identified 9,363 vOTUs in the Shanghai cohort and 10,784 vOTUs in the Beijing cohort as potential CKD-associated vOTUs with a fold-change of ≥ 1.2 and q-value ≤ 0.2. Then, to test the consensus of two independent tests for each vOTUs, the combined p-value was used to further identify meaningful vOTUs associated with the CKD via the *sumlog* function in the R *metap* package. In total, 4,649 vOTUs that exhibited significant differences (combined p-value < 0.001)were considered as the final CKD-associated vOTUs.

*The bacterium-dependency of CKD-associated vOTUs.* To explore the interaction network between CKD-associated vOTUs and bacteria, we categorized these CKD-associated vOTUs into bacterium-dependent and bacterium-independent groups based on virus-host predictions and statistical correlations with 1,303 gut prokaryotic species. Three methods were utilized to evaluate whether there is a relationship between CKD-associated vOTUs and prokaryotes: 1) host assignment mentioned above reported 4,486 phage-host pairs; 2) SparCC 56 co-abundance relationships were established based on the read count profiles of vOTUs and prokaryotes using fastspar v0.0.10 57 with the option “--iterations 20”, where SparCC p-values were determined via 1,000 bootstraps; and 3) co-occurrence relationships were assessed based on the contingency table using Fisher's exact test via the function *fisher.test* in the R platform.

*Functional comparison of gut virome.* We focused on auxiliary metabolic genes (AMGs) of the CKD-associated viruses. According to the method provided by the previous study 43, potential AMGs were manually annotated based on the KEGG database. The occurrence rate of each AMG was calculated as the ratio of vOTUs containing that AMG to the total number of CKD-enriched or HC-enriched vOTUs. Differences in occurrence rates between groups were analyzed using the function *fisher.test*, with p-values adjusted using the function *fdrtool*. Gene arrow maps of vOTU were visualized using the R packages *ggplot2* and *gggenes*.

*Performance of classification models*. We built the random forest model to classify CKD status based on vOTU-level profiles from two cohorts via the function *randomForest*. For each cohort, 70% of the samples were randomly selected as the training set, and the remaining 30% of the samples were used as the testing set. The classification performance of the model was assessed by the area under the receiver operator characteristic curve (AUC) via the function *roc*. Additionally, this process was repeated 10 times with different random splits, and the average AUC from these 10 iterations was used as the final measure of model performance. We also built a model to evaluate the classification performance of CKD viral signatures on other disease states. Specifically, the random forest model was trained using the profiles of CKD viral signatures from 425 CKD patients and 290 healthy controls in this study. The model was then used to predict the case/control status of samples in various public datasets. The classification performance was again evaluated using AUC.

### Analysis of the public faecal metagenomic datasets

For the Chinese populations, we downloaded the publicly available faecal metagenomic datasets from 9 studies, including atherosclerotic cardiovascular disease (ACVD) 58, diabetic kidney disease (DKD, which also classified as CKD patients with eGFR < 60 mL/min/1.73m^2^) 59, colorectal cancer (CRC) 60, hypertension 61, inflammatory bowel disease (IBD) 33, 62, liver cirrhosis (LC) 63, obesity 64, rheumatoid arthritis (RA) 65, and type 2 diabetes (T2D) 66 from the NCBI-SRA (Sequence Read Archive) and EBI (European Bioinformatics Institute) databases. ACVD, hypertension, T2D, and obesity are common complications or primary conditions associated with CKD, while CRC, IBD, LC, and RA have recently been extensively studied in relation to the gut virome and are significant diseases with high prevalence in the population. For other CRC faecal metagenomes, we also downloaded the datasets from 3 European studies 67-70, a USA study 71, and a Japanese study 72. All these faecal metagenomes were quality-controlled and follow-up processed using the same pipeline as the samples of this study.

## Results

### Metagenomic delineation of the gut viral community

This study included faecal samples from two independent cohorts representing a total of 425 CKD patients and 290 HCs that were characterized in previous research 19, 20. Deep whole-metagenomic shotgun sequencing of faeces generated 8.8 Tbp of data (12.3±2.0 Gbp per sample) for exploring gut viral communities. Metagenomic assembly of each faecal metagenome produced a total of 4.82 million long contigs (≥5 kb; total length 95.5 Gbp; Table S1), of which 2.6% (n = 125,332) were recognized as credible viral sequences using both homology-based 43, 44 and feature-based 45 methodologies, alongside 45,427 proviruses identified using the CheckV algorithm 44. These viruses and proviruses were clustered at the species level (>95% nucleotide similarity 73, 74) to generate a catalogue of 46,011 vOTUs (average length: 20,958 bp; N50 length: 35,355 bp; Figure S1A). We compared these vOTUs with two large-scale human gut virus collections, the Gut Virome Database (GVD) 22 and Gut Phage Database (GPD) 23, which contain 32,300 and 71,868 nonredundant vOTUs, respectively. Only 19.4% and 31.6% of the vOTUs in our catalogue were shared with the GVD and GPD, respectively (Figure S1B). The proportions of high-completeness and high-confidence viruses in our catalogue were almost equal to those in the GVD but significantly less than those in the GPD (Figure S1C-D). However, the proportion of low-contamination viruses was remarkably high in both our catalogue (98.0%) and the GVD (94.6%) when compared with that in the GPD (83.4%) (Figure S1E). These findings suggested substantial novelty alongside the high credibility of the gut virome in our dataset. Finally, to facilitate universality, we merged the GVD/GPD-shared viruses into our catalogue and generated 45,849 vOTUs (average length: 27,623 bp; N50 length: 45,002 bp; Figure S1A) for follow-up analysis. The merged catalogue contained 23.3% high-completeness (>90% completeness) and 16.0% medium-completeness (50-90% completeness) vOTUs (Table S2).

**Figure 1:**
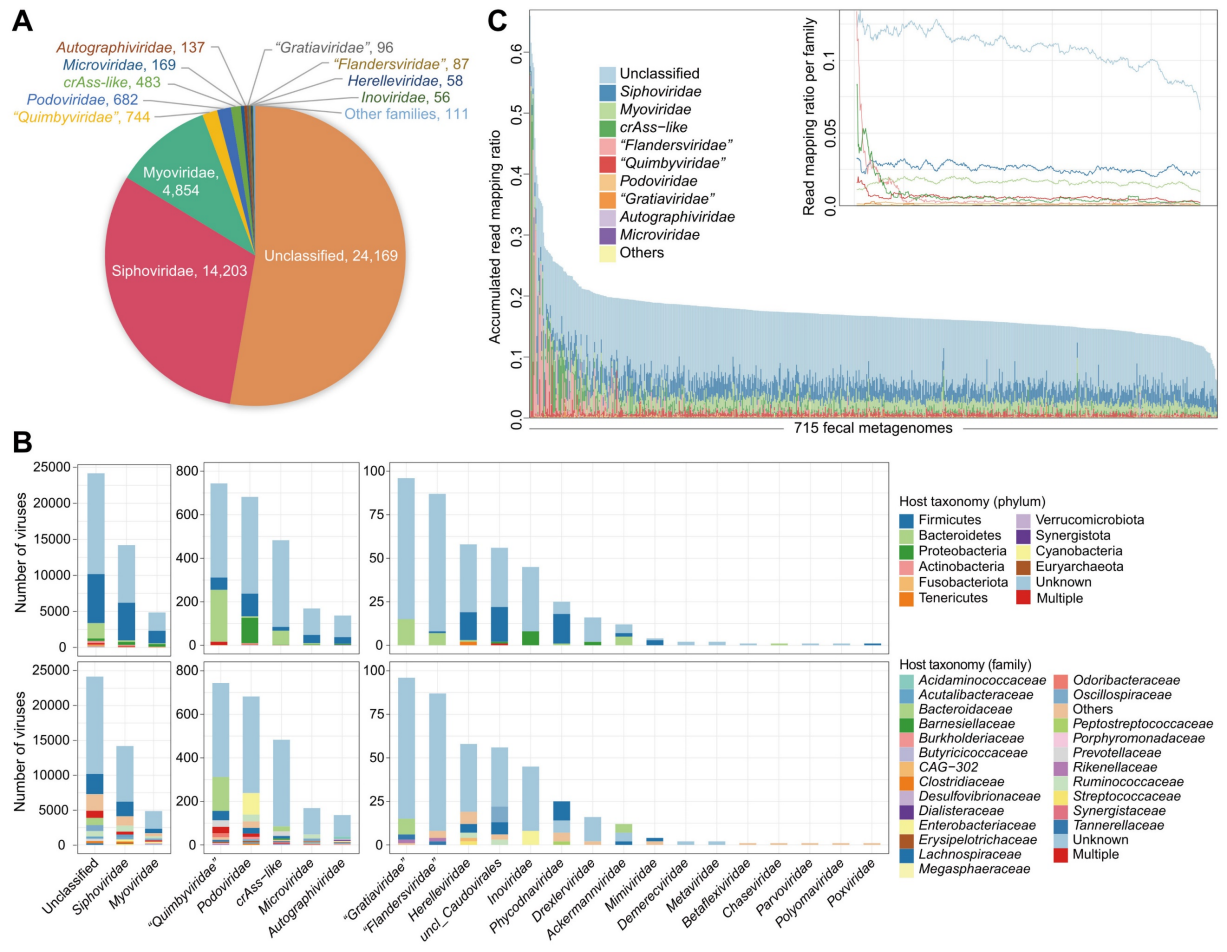
** Summary of taxonomic annotation, host prediction, and metagenomic profiling of the vOTU catalogue constructed from faecal metagenomes. (A)** The number of vOTUs that are assigned into viral taxa at the family level. **(B)** Distribution of prokaryotic hosts of the vOTU catalogue. The vOTUs are grouped at the family level, and the host taxa are showed at the phylum (upper panel) and family (bottom panel) levels. The number of vOTUs that had more than one predicted host is labeled by red color. **(C)** Proportion of metagenomic reads mapped into the vOTU catalogue. Individuals demonstrate a wide range of compositional variations in their gut virome. Inset shows the read proportions for several most abundant families.

A total of 47.3% of the nonredundant vOTUs were robustly assigned to known viral families. *Siphoviridae* (31.0%) and *Myoviridae* (10.6%) constituted the vast majority of taxonomically assigned vOTUs (Figure 1A), and the other representatives included *Podoviridae*, *Microviridae*, *Autographiviridae*, and some eukaryotic viruses (e.g., *Phycodnaviridae*). Notably, 483 vOTUs were classified as *crAss-like* viruses, distinct from other *Podoviridae* members due to unique genomic features 75. Additionally, three new candidate families, including *“Quimbyviridae”, “Gratiaviridae”*, and *“Flandersviridae”*, that were recently identified from the human gut virome 52 were also frequently present in our catalogue.

A total of 42.6% of the 45,849 vOTUs could be assigned to one or more prokaryotic hosts based on their homology to genome sequences or CRISPR spacers of the microbial genomes reconstructed from original faecal metagenomes (representing over 19,000 high-completeness genomes of 1,303 bacterial or archaeal species 54, with the archaea regarded as bacteria for simplicity unless specifically mentioned). The most common identifiable hosts of *Siphoviridae* and *Myoviridae* members were Firmicutes species (mainly *Lachnospiraceae* and *Ruminococcaceae*), while the major hosts of *crAss-like* viruses, *“Quimbyviridae”*, *“Gratiaviridae”*, and *“Flandersviridae”* were Bacteroidetes species, and the hosts of *Podoviridae* members were generally Proteobacteria (mainly *Enterobacteriaceae*) and some Firmicutes species (Figure 1B; Table S3). Only 1.9% (379/19,545) of annotated vOTUs had hosts from multiple bacterial phyla, and only 8.7% (1,709/19,545) of the vOTUs had hosts across different families (Figure S2), suggesting a narrow host range of most gut viruses.

We profiled the viral composition of the faecal samples by mapping metagenomic reads to the vOTU catalogue. On average, 17.4% of reads (ranging from 6.2% to 66.0%) could be robustly mapped into the catalogue (Figure 1C). Nearly half of these viral reads appeared to derive from proviruses, as they mapped in parallel to the bacterial genomes; however, the virus-specific read mapping rate still reached an average of 8.3% in the samples. To ensure accuracy, we investigated 15 samples with the highest proportion (>30%) of viral reads and found that all of these samples were dominated by high-confidence vOTUs, including 2 samples with up to 40-50% of reads aligned to *crAss-like* viruses (Figure S3). An extremely high abundance (up to 95%) of *crAss-like* viruses was also reported in adult gut metagenomes by previous studies 51, 75, 76. Thus, our findings suggested a considerably high or even predominant viral content in the human gut.

### Diversity and structure of the gut virome associated with CKD

To illustrate alteration in the gut viral community associated with CKD, we conducted comparative analyses of virome diversity and compositional structure between CKD patients and HCs across two independent cohorts: Shanghai (171 patients vs. 111 controls) and Beijing (254 patients vs. 179 controls). First, we found that the CKD patients exhibited a lower viral richness (estimated by the observed number of vOTUs) than HCs. However, viral evenness (measured by Shannon and Simpson diversity indexes) did not differ significantly between the two groups (Figure S4A). Comparison of viral composition at the family level revealed that in both Beijing and Shanghai cohorts, the CKD patients were significantly enriched in *Siphoviridae*, *Microviridae*, *Herelleviridae*, and *Drexlerviridae*, while the HCs were more abundant in *Phycodnaviridae* (Figure S4B-C). *“Flandersviridae”* and *“Gratiaviridae”* were markedly enriched in CKD patients in the Beijing cohort and had the same trend in those of the Shanghai cohort, while *Podoviridae* was uniquely enriched in patients of the Shanghai cohort. Conversely, *Myoviridae* exhibited significant depletion in Beijing CKD patients but enriched in those from Shanghai.

**Figure 2:**
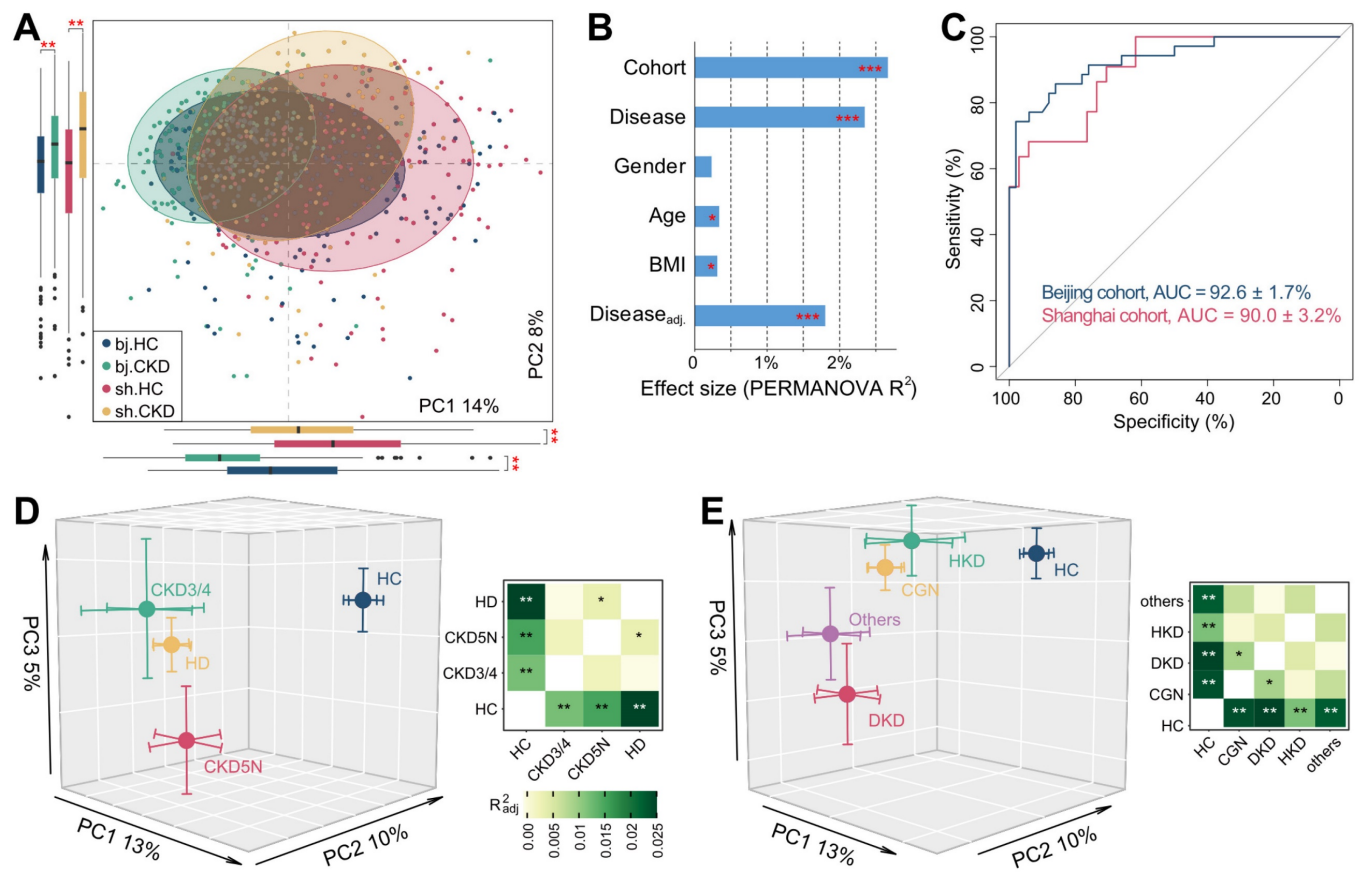
** Structure distinction of gut virome associate with CKD. (A)** PCoA analysis of the Bray-Curtis distances of the gut virome at the vOTU level. Samples are shown at the first and second principal coordinates (PC1 and PC2), and the ratio of variance contributed by these two PCs is shown. Ellipsoids represent a 95% confidence interval surrounding each group. The below and left boxplots show the sample scores in PC1 and PC2 (boxes show medians/quartiles; error bars extend to the most extreme values within 1.5 interquartile ranges). Student's t-test: **, *p <* 0.01. **(B)** PERMANOVA results showing the effect size of phenotype indexes that contribute to the variance of the overall gut virome. Bar plots indicate the explained variation (effect size R^2^) of each phenotype factor. The residual effect size of disease/control stratification after adjusting gender, age, and BMI is also showed. **(C)** Receiver operating characteristic (ROC) analysis of the classification of CKD status using the random forest model. For each cohort, 70% of the samples were randomly selected as the training set, and the remaining 30% of the samples were used as the testing set. The classification performance of the model was assessed by the area under the ROC curve (AUC). The AUC values and 95% confidence intervals (CIs) are shown. **(D-E)** PCoA analysis of the Bray-Curtis distances for the gut virome of samples stratified by their clinical stages (D) and CKD protopathy (E). Samples are shown at the top 3 principal coordinates (PC1, PC2, and PC3), and the ratio of variance contributed by these PCs is shown. The colored circles indicate the center of gravity (mean) of samples for each group, and the error bars indicate the standard errors of the mean. For (D) and (E), the right panels show the pairwise effect sizes among different groups. *Adonis* test with 1,000 permutations: *, *p <* 0.05; **,* p <* 0.01; ***, *p <* 0.001.

PCoA of the gut vOTU profiles showed that the CKD-associated virome significantly deviated from that of controls in both cohorts (Figure 2A). This result was confirmed using PERMANOVA analyses, showing that the CKD status independently explained 2.3% (*adonis P* <0.001) of the overall virome variability. In contrast, confounding factors like age, sex, and BMI explained less than 0.4% of variance each (Figure 2B). Cohort stratification accounted for 2.7% of variance, suggesting that the population and/or geographic factors still exerted considerable influence on the gut virome 28. We next trained two machine learning classifiers to distinguish CKD patients from HCs using the vOTU profiles of the Shanghai and Beijing cohorts separately. Receiver operating characteristic curve analysis showed that both classifiers achieved high discriminatory power with a minimum AUC of 0.90 (Figure 2C). Similar discriminatory ability was also obtained in cross-cohort prediction (Figure S5). These results demonstrated profound changes in the gut virome of CKD patients that could stratify them from HCs.

To test whether the clinical stages of CKD could potentially impact the virome, we classified the patients of the Shanghai cohort into three groups, CKD stages 3-4 (n = 16), CKD stage 5 with haemodialysis (HD, n = 124), and CKD stage 5 without dialysis (CKD5N, n = 31), and compared their vOTU profiles with that of the control population. The viromes changed in a similar fashion in all three patient groups (Figure 2D; Figure S6), likely reflecting the commonalities of the clinical stages. The virome of HD patients showed the farthest distance from that of controls and significantly differed from that of CKD5N patients, suggesting that the dialysis procedure might affect the gut virome. We next evaluated the contribution of underlying diseases to the gut virome by grouping the patients into chronic glomerulonephritis (CGN, n = 102), diabetic kidney disease (DKD, n = 28), hypertensive kidney disease (HKD, n = 14), and other (n = 27) subgroups based on their primary disease types. As expected, all subgroups revealed a similar trend apart from the HC group (Figure 2E; Figure S7A). We also found that the gut virome of DKD patients was most distinct from that of controls and showed a significant deviation from that of CGN patients (*adonis P* = 0.017), which was probably linked to the specific pathogenetic background of DKD patients 77. Random forest-based classifier analysis also showed that the use of the gut virome could identify DKD and non-DKD patients with an AUC of 0.68-0.71 (Figure S7B).

### Identification of CKD-associated viruses in the context of the gut bacterial microbiota

**Figure 3:**
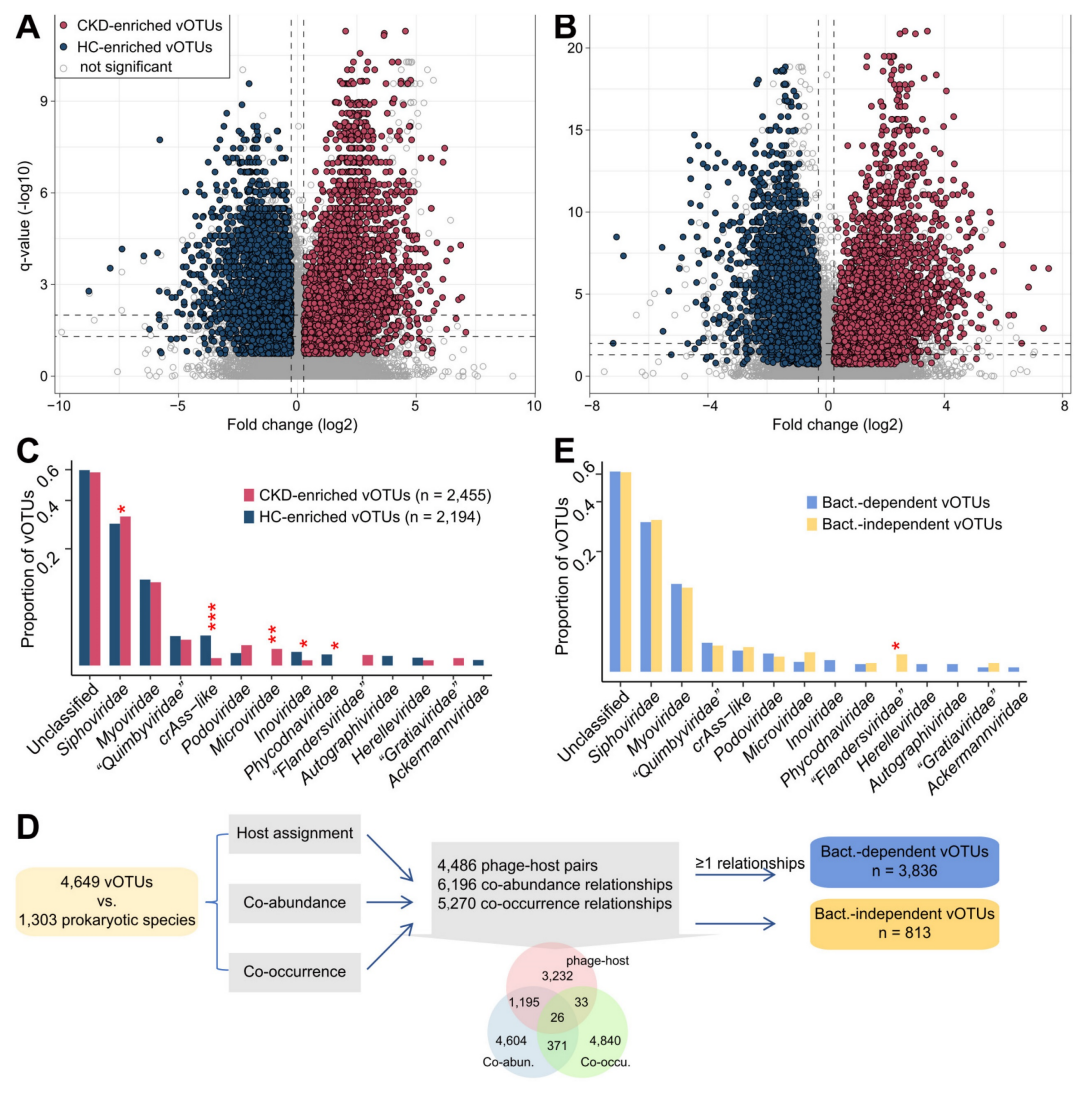
** Identification of CKD-associated vOTUs and exploration of their relationship with bacterial microbiota. (A-B)** Volcano plots showing the fold change vs. *q*-values for all vOTUs in the Shanghai (A) and Beijing (B) cohorts. The X-axis shows the ratio (log2 transformed) of vOTU abundance in CKD patients (fold>0) compared with that in healthy controls (fold<0). The Y-axis shows the *q*-value (-log10 transformed) of a vOTU. The CKD-associated vOTUs with a consistent trend in the two cohorts are shown in red (CKD enriched) and blue (control enriched) circles. Horizontal dotted lines: *q <* 0.05 and *q <* 0.01; vertical dotted lines: fold<-1.2 and fold>1.2. **(C)** Family-level assignment of the CKD-enriched and HC-enriched vOTUs. Fisher's exact test: *, *q <* 0.05; **, *q <* 0.01; ***, *q <* 0.001. **(D)** Workflow for the determination of bacterium-dependency of the CKD-associated vOTUs. The inserted Venn plot shows the overlap of three types of virus-bacterium correlations: host-phage pairs, co-abundance, and co-occurrence. **(E)** Family-level assignment of the bacterium-dependent and bacterium-independent vOTUs. Fisher's exact test: *, *q <* 0.05.

We identified 4,649 differentially abundant vOTUs between CKD patients and healthy subjects using the combined significance level of two independent cohorts (Wilcoxon rank-sum test combined with Fisher's method, *P* <0.005, corresponding to *q* < 0.013; Figure 3A-B). Among these, 2,455 vOTUs were more abundant in CKD patients and 2,194 more abundant in HCs. The majority (81.7% in the Shanghai cohort and 92.6% in the Beijing cohort) of these vOTU abundance differences were also significant within each cohort, and 94.4% of them were still significant after adjusting for sex, age, and BMI (Figure S8A; Table S4). Both CKD-enriched and HC-enriched vOTUs were dominated by members of *Siphoviridae*, *Myoviridae*, and unclassified taxa (Figure 3C). Notably, HC-enriched vOTUs included 29 *crAss-like* viruses, while CKD-enriched vOTUs contained only two; however, CKD patients had 10 *Microviridae* and 4* “Flandersviridae”* members enriched, which were absent in HCs. Notably, the 4,649 CKD-associated vOTUs had a higher detectable rate and relative abundance in the faecal metagenomes than other vOTUs and performed well in distinguishing CKD patients from the controls (Figure S8B-C), highlighting their considerable importance.

Given that the majority of gut viruses are bacteriophages, their lifestyles (e.g., proliferation, migration) typically depend on host microorganisms 23. Such phages might not act on disease independently but rather through certain bacterium-associated mechanisms 39, 78. Accordingly, we examined relationships between 4,649 CKD-associated vOTUs and 1,303 gut prokaryotic species to explore bacterium dependency of these vOTUs for affecting CKD status. Three types of relationships were investigated: 1) the host-phage pairs, 2) co-abundance (defined as SparCC correlation coefficient 56 >0.60, and *q* <0.001), and 3) co-occurrence (defined as Fisher's exact test *q* <0.001). This procedure revealed 3,836 bacterium-dependent vOTUs that had at least one relationship with bacterial species, whereas the remaining 950 vOTUs were bacterium-independent (Figure 3D).

The family-level taxonomic distribution of bacterium-dependent and bacterium-independent vOTUs seemed no different, with the exception of the four CKD-enriched *“Flandersviridae”* vOTUs being independent (Figure 3E). Although some *“Flandersviridae”* viruses are known to infect Bacteroidetes 52 (also see Figure 1B), these 4 vOTUs lacked a host or strong correlation with any bacterial species. Interestingly, we found that two of these *“Flandersviridae”* vOTUs encoded a bacterioferritin gene that was completely absent from other CKD-associated vOTUs (Figure S9A), probably related to their adaptation to the gut environment 79. Next, we performed a comparison of the viral functions of bacterium-dependent and bacterium-independent vOTUs based on the KEGG database and found that their functional contents were visibly different (Figure S9B). Forty-three enzymes were more widespread in the bacterium-independent vOTUs, and 5 enzymes were encoded more frequently in the bacterium-dependent vOTUs (Figure S9C); subsequent analysis based on these differentially abundant enzymes may provide insights into the mechanisms of environmental adaptation and pathogenicity of the bacterium-independent viruses.

### Crosstalk between CKD-associated viruses and bacteria

**Figure 4:**
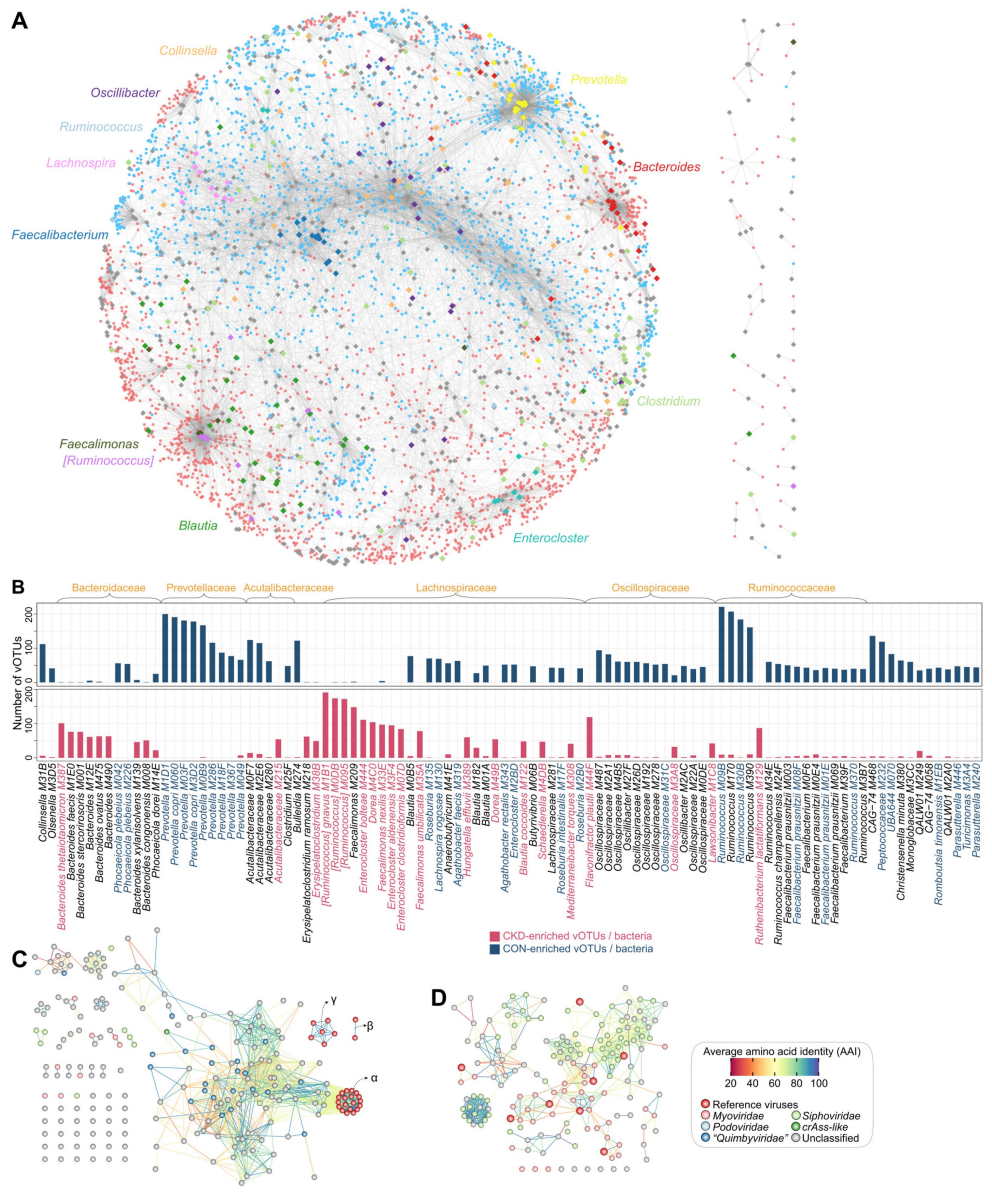
** Network analyses untangle the connections between CKD-associated vOTUs and bacteria. (A)** A virus-bacterium network based on the relationships between 3,836 bacterium-dependent vOTUs and their related bacteria. Small circle nodes represent the vOTUs, and their colors indicate the enrichment: red, CKD-enriched; blue, HC-enriched. Large rhombus nodes represent the bacterial species, and some most frequent species are colored according to their genus-level taxonomic information. Lines connect the vOTUs and bacterial species that have phage-host or co-abundance/occurrence relationships. **(B)** Barplots showing the number of vOTUs correlated with bacterial species. The top 100 species with the largest number of connections are shown. The taxonomic names of bacteria are colored by their enrichment directions: red, CKD-enriched; blue, HC-enriched; black, not significantly differed.** (C-D)** Network showing the genetic distances between *Bacteroides*-connected vOTUs and existing phages (C) and between *Faecalibacterium*-connected vOTUs and existing phages (D). Nodes represent viruses colored by their taxonomic assignment, and lines show the average amino acid identity between two viruses. For (C), three subclades of the existing *B. thetaiotaomicron*-infecting phages are labeled.

We identified a large network of relationships between 3,836 bacterium-dependent vOTUs and their related bacteria (Figure 4A). Within each bacterial family, we found that the host-linked and statistically associated viruses exhibited high similarity in taxonomic assignments (Figure S10). Considering that a large number of potential host-phage pairs could not be identified by current technology but might be identified as co-abundance/occurrence relationships, this result indicated that host-phage affiliation was the major driver of the virus-bacterium interaction network.

The CKD-enriched vOTUs were dominantly connected to bacterial members that belonged to *Bacteroides*, *[Ruminococcus]*, *Faecalimonas*, *Enterocloster*, *Dorea*, and *Erysipelatoclostridium*, followed by several species, including *Flavonifractor plautii*, *Ruthenibacterium lactatiformans*, and *Hungatella effluvia* (Figure 4B). Many of these species, such as *[Ruminococcus] gnavus*, *Faecalimonas nexilis, Enterocloster bolteae*, *Enterocloster clostridioformis*, and *Flavonifractor plautii*, have been identified as potentially harmful bacteria in human diseases (see Discussion), and consistently, most of these virus-related species have been found to be significantly overabundant in the gut bacteriome of CKD patients 54. Viruses that correlated to *Bacteroides* spp. were particularly noticed because *Bacteroides*-infecting phages have been reported as the most abundant viruses in the human gut and might contribute to metabolic disorders 80, 81. Twenty-six *Bacteroides* species had targeted to 207 vOTUs (including 159 CKD-enriched ones) in the virus-bacterium network, with *B. thetaiotaomicron* being the most frequently associated (Figure S11A). Twenty-two of 207 vOTUs belonged to the *“Quimbyviridae”* family, while most others were unknown taxonomy. Moreover, genome-level homology analysis of 207 vOTUs and known *B. thetaiotaomicron*-infecting phages 82 showed that nearly all of these vOTUs were newly identified (Figure 4C; Figure S12). These findings suggested largely unexplored phage diversity in the gut virome and raised further research on their relevance to CKD. In addition, some CKD-enriched vOTUs were linked to two crucial uraemic toxin-producing clades,* Eggerthella* and *Fusobacterium* 20, and showed direct positive correlations with the serum concentrations of several toxins in the individuals of the two cohorts (Figure S13), suggesting that they probably affect the toxin production process in the human gut.

In contrast, HC-enriched vOTUs were frequently connected to the bacterial members of *Prevotella*, *Ruminococcus*, *Faecalibacterium*, *Oscillospiraceae*, *Roseburia*, *Blautia*, *Acutalibacteraceae*, and other taxa (Figure 4C). *Prevotella* spp. were the most prevalent members, connecting 363 vOTUs (311 of which were HC-enriched) in the virus-bacterium network, including 22 *crAss-like* and 21 *“Quimbyviridae”* viruses and many taxonomically unknown viruses (Figure S11B). Previous studies have widely validated that the depletion of *Prevotella* spp. was associated with CKD and ESRD 83. Our results thus suggested that *Prevotella* phages could also be noted for their potential roles in CKD or related diseases. Another typical bacterial clade was *Faecalibacterium*, which contained 15 species in the virus-bacterium network (Figure S11C), including the widely reported butyrate-producing probiotic *F. prausnitzii* 84, 85. The *Faecalibacterium*-related viruses (n=155 vOTUs, including 133 HC-enriched vOTUs) were concentrated in *Siphoviridae* and *Myoviridae*, and most of them (138/155) were novel viruses compared with the known *Faecalibacterium* phages 86 (Figure 4D); the potential roles of these viruses in the CKD gut virome are worth future exploration. In addition, several other species connected to numerous HC-enriched vOTUs were also butyrate producers, including *Roseburia* (mainly *R. intestinalis*), *Butyrivibrio*, and *Lachnospira* spp. 87, 88.

### Functions of the CKD-associated viruses

To explore the functional and metabolic capabilities of the CKD-associated viruses, we predicted a total of 221,424 protein-coding genes from 4,649 vOTUs and annotated the functions of 17.3% of these genes by searching against the KEGG database. Most genes were categorized under typical viral functions such as DNA replication and repair, transcription, and prokaryotic defence system, with no significant deviation observed between CKD-enriched and HC-enriched vOTUs (Figure S14A). We next specifically focused on viral AMGs since phage-encoded AMGs are known to redirect host functional capacities, thereby directly influencing bacterial ecology 39, 89. A total of 10,376 genes were identified as viral AMGs based on a previously curated list 43, which composed 27.1% of the annotated genes (4.7% of total genes) of the CKD-associated vOTUs. These AMGs were primarily linked to genetic information processing, particularly nucleotide (purine and pyrimidine) metabolism and peptidases and inhibitors (Figure S14B). Notably, genes related to peptidoglycan biosynthesis and degradation were frequently encoded by the viral genomes, with CKD-enriched vOTUs containing a higher proportion than HC-enriched vOTUs (5.9% vs. 4.5% of respective AMGs; Fisher's exact test* q*=0.002). Furthermore, genes involved in sulfur metabolism were also prevalent in vOTUs, in agreement with recent reports showing that human gut viruses actively participate in both organic and inorganic sulfur metabolism 90, 91; these genes accounted for similar proportions between the CKD-enriched and HC-enriched vOTUs (4.5% vs. 4.7%, *q*=0.409).

**Figure 5:**
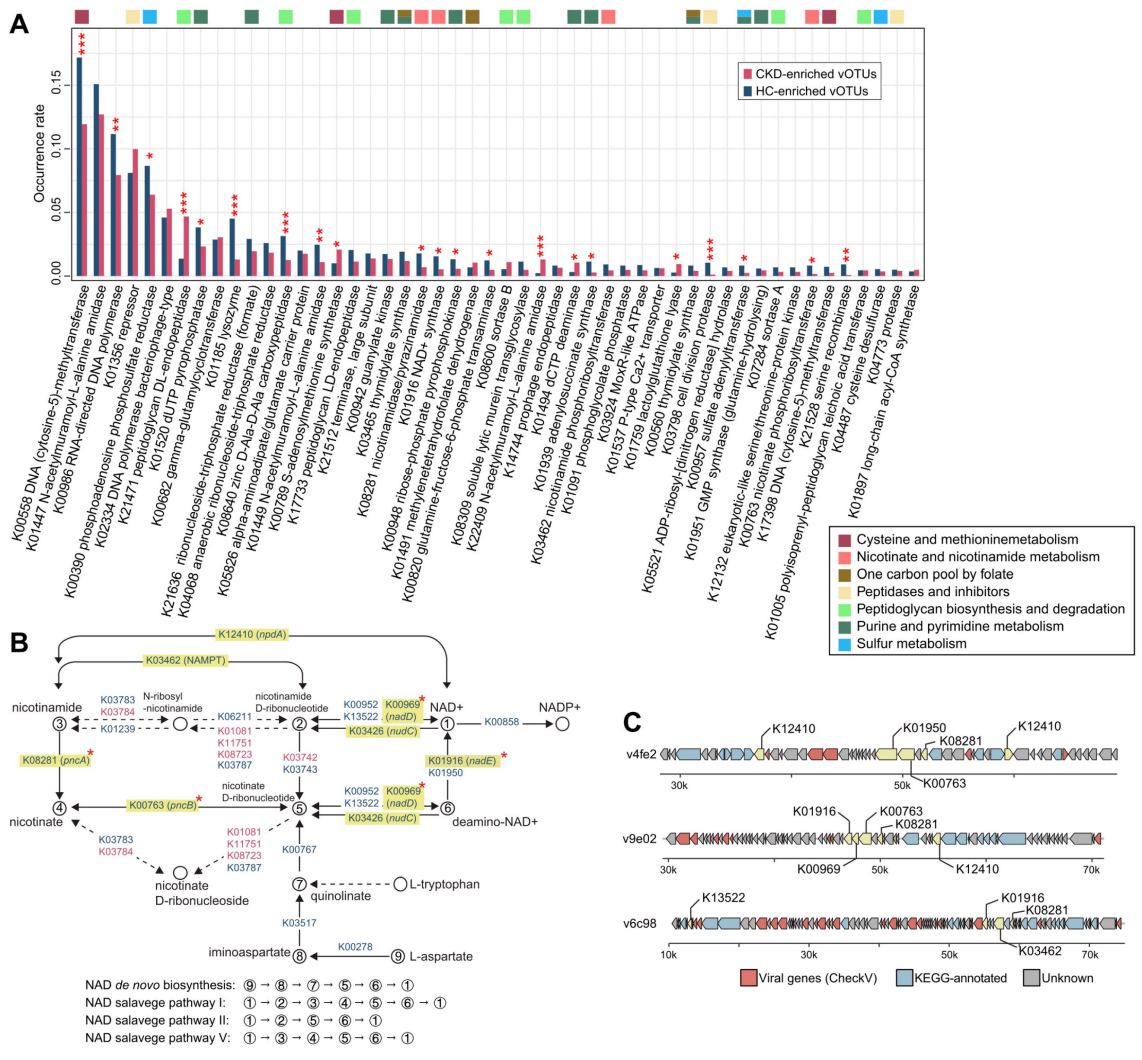
** Functional differences between the CKD-enriched and HC-enriched vOTUs. (A)** Occurrence rate of 50 most frequent AMGs. The functional categories of each AMG are shown by colored squares. Fisher's exact test: *, *q <* 0.05; **, *q <* 0.01; ***, *q <* 0.001. **(B)** Enzymes and pathways involving NAD^+^ *de novo* biosynthesis and salvage. This diagram is exacted from the KEGG pathway database with manual modifications. Enzymes are colored by their enrichment directions (red, CKD-enriched; blue, HC-enriched) and six key enzymes in the pathways are labeled by yellow boxes. Fisher's exact test: *, *q <* 0.05. **(C)** Representative alignment of three viral contigs encoding multiple genetic contexts. The viral genes of the contigs are identified by CheckV.

We compared the occurrence rate of AMGs between CKD-enriched and HC-enriched vOTUs at the enzyme level (representing 1,316 auxiliary metabolic enzymes based on the KEGG database; Table S5). The most frequent AMG was a DNA-cytosine methyltransferase (K00558), which mediates cytosine DNA methylation using S-adenosylmethionine (SAM) as a methyl donor 92; this enzyme was found to be more prevalent in HC-enriched vOTUs compared to CKD-enriched vOTUs (occurrence rate 17.3% vs. 11.9%; Fisher's exact test* q*<0.001). Interestingly, 21 of the 50 most frequent AMGs showed significant differences in frequency between the two groups (Figure 5A). HC-enriched vOTUs had a higher frequency of enzymes such as K00986 (RNA-directed DNA polymerase), K01520 (dUTP pyrophosphatase), K01185 (lysozyme), and two enzymes involving assimilatory sulfate reduction (K00390 [phosphoadenosine phosphosulfate reductase] and K00957 [sulfate adenylyltransferase]) compared to CKD-enriched vOTUs. In contrast, enzymes such as K21471 (peptidoglycan DL-endopeptidase), K00789 (SAM synthetase), and K22409 (N-acetylmuramoyl-L-alanine amidase) were more frequent in CKD-enriched vOTUs. Although it is outside the scope of this study to investigate the mechanism of all differentially abundant AMGs, we explored the prominent example of the enzymes involved in nicotinamide adenine dinucleotide (NAD^+^) biosynthesis. From the CKD-associated vOTUs, we identified 25 enzymes involved in several pathways of NAD^+^ *de novo* biosynthesis and salvage (Figure 5B), most of which were more likely to be encoded by HC-enriched vOTUs than by CKD-enriched vOTUs. In particular, 4 critical enzymes of the pathways, namely K01916 (NAD^+^ synthase, participating in the NAD^+^ *de novo* biosynthesis pathway), K00969 (nicotinate-nucleotide adenylyltransferase, participating NAD^+^ salvage pathways I and II), and K08281/K00763 (*pncAB*, participating salvage pathways I and V), were significantly higher in frequency in HC-enriched vOTUs (Figure S15A). Genome analysis further confirmed that these enzymes were usually encoded in multiple genetic contexts within HC-enriched vOTUs (Figure 5C; Figure S15B). Additionally, a comparison of gene expression in faecal metagenomes revealed a higher abundance of NAD^+^ biosynthesis-associated enzymes in HCs compared to CKD patients (Figure S16). Collectively, these findings suggest a higher NAD^+^ synthesis capacity in HC-enriched vOTUs as well as in the gut virome of HCs. Further investigations based on well-designed clinical and/or animal experiments will ultimately elucidate the mechanism underlying the interaction between this vital function and CKD aetiology.

### CKD viral signatures correlate with common diseases

**Figure 6:**
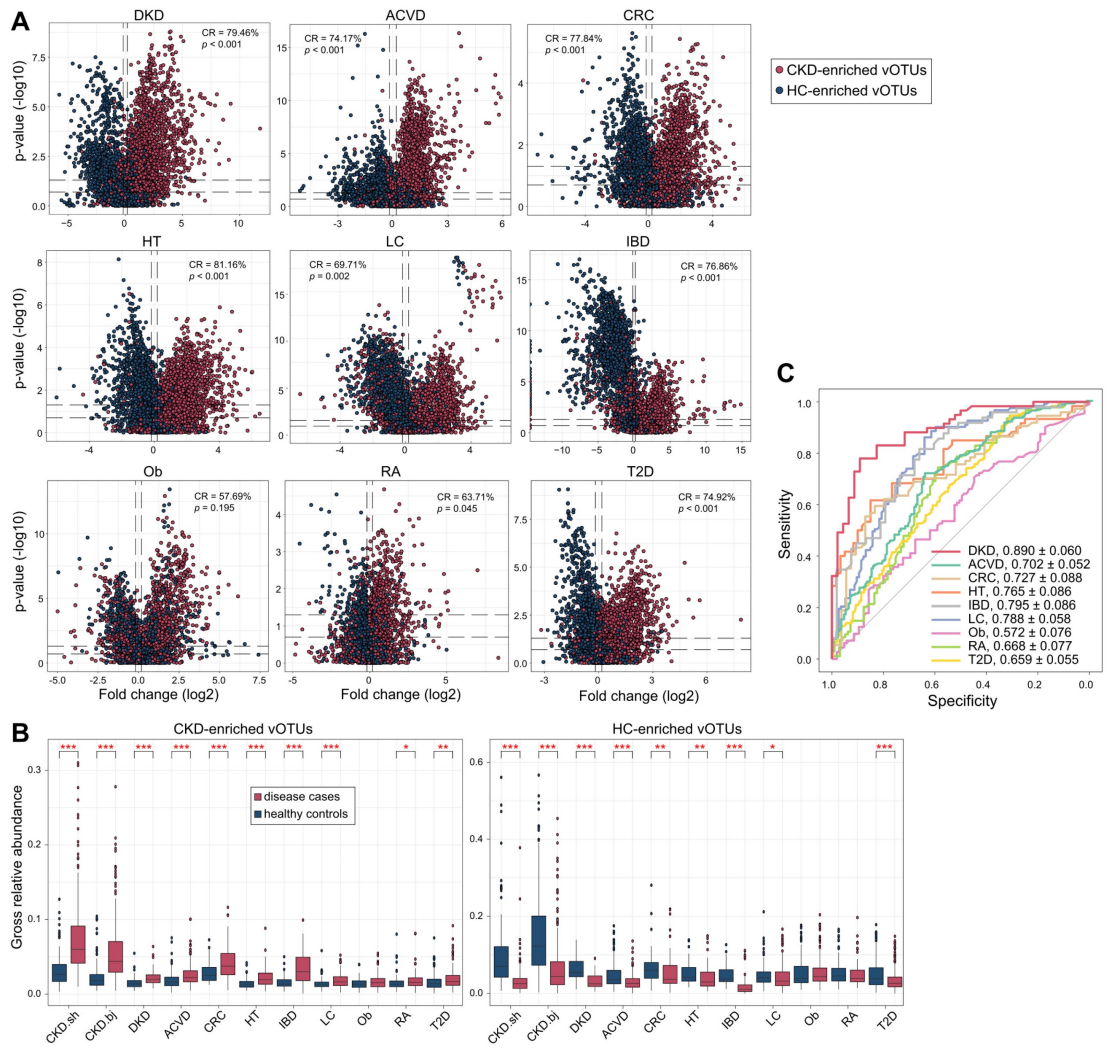
** Alterations of CKD-associated vOTUs in other diseases. (A)** Volcano plots showing the fold change vs. *q*-values for vOTUs among 9 studies. The X-axis shows the ratio (log2 transformed) of vOTU abundance in disease cases (fold>0) compared with that in healthy controls (fold<0). The Y-axis shows the *q*-value (-log10 transformed) of a vOTU. vOTUs are colored by their enrichment directions in CKD patients vs. healthy controls. Horizontal dotted lines: *q <* 0.05 and *q <* 0.01; vertical dotted lines: fold<-1.2 and fold>1.2. **(B)** Boxplots show the gross relative abundance of CKD-associated vOTUs in the gut virome of subjects from 9 studies. Boxes represent the interquartile range between the first and third quartiles and the median (internal line). Whiskers denote the lowest and highest values within 1.5 times the range of the first and third quartiles, respectively; dots represent outlier samples beyond the whiskers. Student's t-test: *, *q <* 0.05; **, *q <* 0.01; ***, *q <* 0.001. **(C)** Receiver operating characteristic (ROC) analysis of the classification of case/control status using the random forest model trained by 425 CKD patients and 290 healthy controls. The classification performance of the model was assessed by the area under the ROC curve (AUC). The AUC values and 95% confidence intervals (CIs) are shown.

To test the specificity and universality of CKD viral signatures, we curated the faecal metagenomes previously studied for microbial alterations in common diseases and explored the changes in CKD-associated vOTUs in them. In total, 1,901 available faecal metagenomes (993 cases and 908 controls, Table S6) covering 9 different diseases were downloaded and processed using a standardized pipeline (see Materials and Methods). All data were sourced from Chinese cohorts to minimize the impact of inter-country variations in gut viromes 28. Using this large cohort, we quantified the relative abundances of 4,649 CKD-associated vOTUs and compared them between cases and controls for each disease. For all 9 cohorts, the majority (ranging from 86.8% to 99.4%) of vOTUs were frequently detected in over 20% of individuals (Figure S17), suggesting that CKD-associated vOTUs are widespread in the human gut virome regardless of region and disease status. For each disease, we calculated a “consistency rate”, defined as the proportion of vOTUs exhibiting a consistent trend in mean abundance between cases and controls compared with the observation in CKD patients vs. controls. Strikingly, the consistency rate was markedly high in the cohorts for ACVD, CRC, DKD, hypertension, IBD, and T2D, ranging from 74.2% in the ACVD cohort to 81.2% in the hypertension cohort (permutated *P* <0.001 for all diseases) (Figure 6A). In contrast, consistency rates in the RA and obesity cohorts were lower, at 63.7% (permutated *P* =0.045) and 57.7% (permutated *P* =0.195), respectively. Furthermore, the gross abundances of CKD-enriched vOTUs were significantly higher in the patients with ACVD, CRC, DKD, hypertension, IBD, LC, RA, and T2D than in controls, while the gross abundances of CKD-depleted vOTUs were also significantly reduced in these patients, except in the RA cohort (Figure 6B). Combining these findings suggested that the CKD viral signatures are highly reproducible in patients with ACVD, CRC, DKD, hypertension, LC, and T2D, and partly reproducible in RA patients, but do not appear to be associated with obesity. In addition, we assessed CKD viral signatures in CRC-associated faecal microbiomes from European, USA, and Japanese populations (consisting of 6 studies with 248 patients and 448 controls) and found that most of these signatures were reproducible (median consistency rate 66.2%; permutated *P* <0.05 in 4 of 6 cohorts; Figure S18; Table S6), supporting the hypothesis that CKD and CRC may share common signatures within their associated gut viromes.

Using the abundance of CKD-associated vOTUs in patients and healthy individuals in the current study, we trained a machine learning classifier to distinguish cases and controls for each disease cohort. This model achieved the highest AUC of 0.890 for distinguishing DKD patients from controls, and it also demonstrated considerable discriminatory power for predicting ACVD, CRC, hypertension, IBD, and LC, with AUCs ranging from 0.702 to 0.795 (Figure 6C). These findings underscore the impressive diagnostic potential of CKD viral signatures in the external kidney disease cohort as well as in other related diseases.

## Discussion

In addition to multiple epidemiological factors being associated with CKD, the gut microbiota remains an important aspect that likely influences CKD etiopathogenesis and development 83. Given the vast diversity of viruses within the human gut microbiome and their impact on host health and disease 23, 93, we hypothesize that this often-overlooked component may be pertinent to CKD. Our study is the first to identify gut virome signatures associated with CKD, paving the way for further mechanistic investigations.

To establish the material for virome analysis, we constructed a virus catalogue of 45,849 nonredundant vOTUs derived from 715 deeply sequenced faecal metagenomes based on the methodology developed by recent studies 22, 23, 94, 95. A significant proportion of these vOTUs were newly found compared with the existing human virus databases, likely reflecting the advantage of deep metagenomic sequencing for viral genome recovery 96 or the specific characteristics of the Chinese population. The current virus catalogues included on average 17.4% of the sequencing reads in the original faecal metagenomes, with some samples exhibiting viral read proportions of 40-50%. This finding is remarkably higher than previous estimates (usually <3%) 81, 97, 98, although it aligns more closely with Nayfach *et al*.'s estimation of 8.6% 94, suggesting the presence of an unexplored extensive viral “dark matter” in the human gut. Overall, our virus catalogue and subsequent findings highlight the efficacy of metagenome-based methodology for exploring the complex human gut viral community.

Consistent with previous studies 28, 99, the gut viromes of both CKD patients and HCs were dominated by several families, including *Siphoviridae*, *Myoviridae*, and *Podoviridae* (containing *crAss-like* viruses), all belonging to the dsDNA virus order *Caudovirales*. *Siphoviridae* and *Microviridae* (a ssDNA virus family that is prevalent in the human gut) were significantly enriched in CKD patients. While most members of these two families are temperate viruses 100, 101, their specific functions in the CKD virome remain unclear. Another prominent CKD-enriched family was *“Flandersviridae”*, which contained 4 vOTUs consistently enriched in CKD patients. This recently described viral taxon is highly abundant and widespread in the human gut, suspected to be an obligately lytic virus 52, 102. Genome analysis of these 4 *“Flandersviridae”* vOTUs showed that they uniquely encoded a bacterioferritin gene, though their relevance to CKD is still under investigation. Importantly, all 4 *“Flandersviridae”* vOTUs were independently associated with CKD, regardless of the gut bacterial community, suggesting that they might act on CKD status in a certain way that warrants future exploration. To further investigate the CKD-associated vOTUs, we connected them to gut bacteria using both host-phage interactions and statistical relationships. CKD-enriched vOTUs were frequently connected to some widely reported harmful bacteria, such as *B. thetaiotaomicron* (a gut commensal that probably promotes enteric infections and obesity 64, 103),* [Ruminococcus] gnavus* (a proinflammatory bacterium associated with multiple diseases 104, 105), *Erysipelatoclostridium ramosum* (formerly *Clostridium ramosum*, an obesogenic bacterium 106, 107), *Enterocloster bolteae/clostridioformis* (opportunistic pathogens previously included in the Clostridium XIVa group 108), and *Flavonifractor plautii* (a flavonoid-degrading bacterium involved in CRC and uraemic toxin production in CKD patients 20, 109). Conversely, HC-enriched vOTUs were generally connected to beneficial species, including *Blautia* (one of the most dominant gut microbial taxa with probiotic properties such as biological transformation and metabolic syndrome alleviation 110), *Faecalibacterium*, and *Roseburia* members. Given that an extensive number of correlations existed between CKD-associated vOTUs and these species, our findings suggested that the viruses may interact with bacteria to further influence disease progression.

To study the functional contents of gut viruses and their relevance to CKD, we functionally annotated the viral genes and performed a comparative analysis between CKD-enriched and HC-enriched vOTUs. The majority of the annotated viral genes represented typical viral functions, such as DNA replication/repair and nucleotide biosynthesis, in accordance with previous observations 44. Viral AMGs were especially focused on because they can be actively expressed during infection to reprogram host metabolism and provide viruses with fitness advantages 43, 111. A considerable proportion (>5%) of AMGs were involved in peptidoglycan biosynthesis and degradation, with these genes being more abundantly encoded by CKD-enriched vOTUs than HC-enriched vOTUs. Peptidoglycan metabolism genes are also enriched in the gut virome of RA patients who are anti-cyclic citrullinated protein antibody negative, indicating their potential interactions with the immune system 39. The most prominent enzyme of these genes was peptidoglycan DL-endopeptidase (K21471), also known as peptidoglycan hydrolase *cwlO*, with cell wall-degrading activity 112, 113, which showed a several times higher prevalence rate in the CKD-enriched vOTUs than in HC-enriched vOTUs. Virus-encoded peptidoglycan hydrolases are lytic enzymes that locally degrade the peptidoglycan of the bacterial cell wall during infection 114. The heightened production of peptidoglycan hydrolases in CKD-enriched vOTUs suggests a potential shift from lysogenic to lytic replication within the temperate phage population, leading to increased levels of proinflammatory bacterial debris that could influence local innate immune responses and mucosal immune system 115, 116, as previously observed in the gut virome of IBD patients 31. In addition to K21471, several additional viral-encoded genes also showed peptidoglycan hydrolase activity 117, including lysozyme (K01185), peptidoglycan LD-endopeptidase (K17733), and three N-acetylmuramoyl-L-alanine amidases (K01447, K01449, and K22409). Of these, K22409 was more abundant in CKD-enriched vOTUs, whereas K01185 and K01449 were less frequent; subsequent studies of these enzymes will help untangle the connections between virus-mediated peptidoglycan degradation, bacterial lysis regulation, and CKD pathogenesis.

Another striking finding was the lower frequency of genes involved in NAD^+^ biosynthesis in CKD-enriched vOTUs compared to HC-enriched vOTUs, coupled with reduced expression in the gut virome of CKD patients. Various studies have verified the presence of NAD^+^ synthesis genes in viruses 118, 119 and revealed their indispensable role in phage DNA replication and exploitation of host metabolic pathways and biochemical processes during viral infection 120. The impact of reduced NAD^+^ synthesis in the virome of CKD patients is unknown and is probably linked to viral reproduction, antioxidation, and other effects. Notably, there have been numerous studies highlighting the disruption of *de novo* NAD^+^ biosynthesis in human cases of acute kidney injury (AKI) 121-123. Researchers have found that in mouse models of AKI, there is a decrease in renal NAD^+^ levels, an increase in quinolinate levels, and a reduction in the activity of quinolinate phosphoribosyltransferase 121. Moreover, the randomized double-blind clinical trial has shown that the treatment aimed at restoring NAD^+^ could constitute a significant advance for patients at risk of AKI 121. Our findings suggest that the gut virome of CKD patients also exhibits a de novo NAD^+^ biosynthetic downregulation, which seems to hint at a broader metabolic dysregulation in these individuals. This observation could open new avenues for understanding the role of viral communities in the pathophysiology of CKD and might point towards potential therapeutic strategies that target the gut virome to restore metabolic balance.

Numerous studies have established associations between gut microbiota and common diseases, often identifying consistent microbiome changes across multiple diseases 124-126. Typically, systemic diseases such as IBD and CRC are marked by the presence of certain opportunistic pathogens 69, 124, whereas cardiometabolic and immune diseases are characterized by a depletion of potentially beneficial microbes (e.g., the aforementioned short-chain fatty acid (SCFA)-producing bacteria) 58, 127. To determine the generalizable scope of this knowledge regarding gut viruses, we profiled the gut virome of 9 available Chinese cohorts with different diseases and found that most CKD-associated viral signatures were reproducible in cohorts with CVD, CRC, DKD, hypertension, IBD, LC, and T2D cohorts. The consistency of virus signatures across these diseases may not only be correlated to their common changes in the bacterial microbiome but also suggest that some viruses can independently influence multiple diseases through a consistent manner, such as immunoregulation or viral infection 128. Our results highlight the importance of a broad exploration of the gut viromes of microbiome-related complex diseases.

Unlike our whole-metagenome-based technology, virus-like particle (VLP) enrichment followed by subsequent metagenomic sequencing (referred to as VLP metagenomic technology) has shown promise in illuminating gut viromes across diseases 129. However, recent studies have revealed that these two technologies substantially differ in efficiency and coverage for viral identification 22, 96, suggesting that a comprehensive “whole virome” in faecal specimens should be measured using both technologies. This represents a critical area for improvement in future studies. CKD is a complex disease with an uncertain aetiology and is often accompanied by various complications, such as hypertension, diabetes, and constipation. However, due to the limited sample size in our study, we did not conduct a specific analysis on the potential impacts of these factors on the virome of CKD patients. Future research should aim to further explore these associations, as a more detailed examination could uncover important insights into how these coexisting conditions may interact with the viral signatures associated with CKD. On the other hand, considering the remarkable effect of disease severity on the gut bacteriome of CKD patients 18, 130, its effect on the gut virome also needs to be explored by future studies.

## Conclusions

By a metagenome-wide analysis of the virome of faecal samples from CKD patients and healthy controls, we revealed that the gut viral community of CKD was substantially altered, occurring across different cohorts, host properties, disease severities and underlying diseases. Our study identified numerous CKD-associated viruses and uncovered their 1) interactions with gut bacteria and 2) specific functions that may be linked to substance metabolism in the gut ecosystem. Overall, this research describes an overview of the gut viral community of CKD patients and HCs and implicates specific viruses in CKD, offering new resources and insights to assess viral involvement in other complex diseases.

## Supplementary Material

Supplementary **Additional file 1: Table S1.** Statistics of data production, metagenomic assembly, and viral contig prediction for 715 samples used in this study. **Table S2.** The CheckV-evaluated result of 45,849 vOTUs. **Table S3.** Detailed information of the vOTU catalogue integrating at the family level. **Table S4.** Detailed information of 4,649 CKD-associated vOTUs. **Table S5.** Detailed information of the viral AMGs occurred in the CKD-enriched and HC-enriched vOTUs. **Table S6.** Detailed information of the external studies.

Supplementary **Additional file 2: Figure S1.** Construction of the vOTU catalogue and comparison with other databases. **Figure S2.** Host assignment of the vOTU catalogue. **Figure S3.** Composition of the samples with the highest proportion of viruses. **Figure S4.** Comparison of alpha diversity indexes and family-level compositions between CKD patients and healthy controls. **Figure S5.** Receiver operating characteristic (ROC) analysis of the classification of CKD status. **Figure S6.** Comparison of gut virome among CKD patients at different clinical stages. **Figure S7.** Comparison of gut virome among CKD patients with different protopathies. **Figure S8.** Identification of CKD-associated vOTUs. **Figure S9.** Functional comparison between the bacterium-dependent and bacterium-independent vOTUs. **Figure S10.** Comparison of host-phage pairs and statistical relationships. **Figure S11.** Virus-bacterium networks for *Bacteroides*, *Prevotella*, and *Faecalibacterium* spp. and their related viruses. **Figure S12.** Genetic distances between *Bacteroides*-connected vOTUs and existing *B. thetaiotaomicron*-infecting phages. **Figure S13.** Correlations between the *Eggerthella*/*Fusobacterium*-related vOTUs and the serum uremic toxin concentrations of individuals. **Figure S14.** Comparison of the functional composition between CKD-enriched and HC-enriched vOTUs. **Figure S15.** Enzymes that involved in NAD^+^ *de novo* biosynthesis and salvage. **Figure S16.** Distribution of 25 NAD^+^ biosynthesis-associated enzymes in the faecal metagenomes of all samples. **Figure S17.** Detection rate of 4,649 CKD-associated vOTUs in 9 cohorts. **Figure S18.** Alterations of CKD-associated vOTUs in 6 studies of CRC faecal microbiome.

## References

[1] Levin A, Ahmed SB, Carrero JJ, Foster B, Francis A, Hall RK (2024). Executive summary of the KDIGO 2024 clinical practice guideline for the evaluation and management of chronic kidney disease: known knowns and known unknowns. Kidney Int.

[2] Bikbov B, Purcell CA, Levey AS, Smith M, Abdoli A, Abebe M (2020). Global, regional, and national burden of chronic kidney disease, 1990-2017: a systematic analysis for the Global Burden of Disease Study 2017. Lancet.

[3] Foreman KJ, Marquez N, Dolgert A, Fukutaki K, Fullman N, McGaughey M (2018). Forecasting life expectancy, years of life lost, and all-cause and cause-specific mortality for 250 causes of death: reference and alternative scenarios for 2016-40 for 195 countries and territories. Lancet.

[4] Kovesdy CP (2022). Epidemiology of chronic kidney disease: an update 2022. Kidney Int Suppl.

[5] Hapca S, Siddiqui MK, Kwan RS, Lim M, Matthew S, Doney AS (2021). The relationship between AKI and CKD in patients with type 2 diabetes: an observational cohort study. J Am Soc Nephrol.

[6] Kalantar-Zadeh K, Jafar TH, Nitsch D, Neuen BL, Perkovic V (2021). Chronic kidney disease. Lancet.

[7] Baker M, Perazella MA (2020). NSAIDs in CKD: are they safe?. Am J Kidney Dis.

[8] Hu L, Napoletano A, Provenzano M, Garofalo C, Bini C, Comai G (2022). Mineral bone disorders in kidney disease patients: the ever-current topic. Int J Mol Sci.

[9] Köttgen A, Cornec-Le Gall E, Halbritter J, Kiryluk K, Mallett AJ, Parekh RS (2022). Genetics in chronic kidney disease: conclusions from a kidney disease: improving global outcomes (KDIGO) controversies conference. Kidney Int.

[10] Romagnani P, Remuzzi G, Glassock R, Levin A, Jager KJ, Tonelli M (2017). Chronic kidney disease. Nat Rev Dis Primers.

[11] Friedman DJ (2019). Genes and environment in chronic kidney disease hotspots. Curr Opin Nephrol Hypertens.

[12] McCarville JL, Chen GY, Cuevas VD, Troha K, Ayres JS (2020). Microbiota metabolites in health and disease. Annu Rev Immunol.

[13] Yang T, Richards EM, Pepine CJ, Raizada MK (2018). The gut microbiota and the brain-gut-kidney axis in hypertension and chronic kidney disease. Nat Rev Nephrol.

[14] Glorieux G, Gryp T, Perna A (2020). Gut-derived metabolites and their role in immune dysfunction in chronic Kidney Disease. Toxins (Basel).

[15] Khoury T, Tzukert K, Abel R, Abu Rmeileh A, Levi R, Ilan Y (2017). The gut-kidney axis in chronic renal failure: a new potential target for therapy. Hemodial Int.

[16] Kanbay M, Onal EM, Afsar B, Dagel T, Yerlikaya A, Covic A (2018). The crosstalk of gut microbiota and chronic kidney disease: role of inflammation, proteinuria, hypertension, and diabetes mellitus. Int Urol Nephrol.

[17] Li F, Wang M, Wang J, Li R, Zhang Y (2019). Alterations to the gut microbiota and their correlation with inflammatory factors in chronic kidney disease. Front Cell Infect Microbiol.

[18] Ren Z, Fan Y, Li A, Shen Q, Wu J, Ren L (2020). Alterations of the human gut microbiome in chronic kidney disease. Adv Sci (Weinh).

[19] Zhang P, Wang X, Li S, Cao X, Zou J, Fang Y (2023). Metagenome-wide analysis uncovers gut microbial signatures and implicates taxon-specific functions in end-stage renal disease. Genome Biol.

[20] Wang X, Yang S, Li S, Zhao L, Hao Y, Qin J (2020). Aberrant gut microbiota alters host metabolome and impacts renal failure in humans and rodents. Gut.

[21] Ren Y, Chen L, Guo R, Ma S, Li S, Zhang Y (2024). Altered gut mycobiome in patients with end-stage renal disease and its correlations with serum and fecal metabolomes. J Transl Med.

[22] Gregory AC, Zablocki O, Zayed AA, Howell A, Bolduc B, Sullivan MB (2020). The gut virome database reveals age-dependent patterns of virome diversity in the human gut. Cell Host Microbe.

[23] Camarillo-Guerrero LF, Almeida A, Rangel-Pineros G, Finn RD, Lawley TD (2021). Massive expansion of human gut bacteriophage diversity. Cell.

[24] Canchaya C, Fournous G, Chibani-Chennoufi S, Dillmann ML, Brussow H (2003). Phage as agents of lateral gene transfer. Curr Opin Microbiol.

[25] Barr JJ, Auro R, Furlan M, Whiteson KL, Erb ML, Pogliano J (2013). Bacteriophage adhering to mucus provide a non-host-derived immunity. Proc Natl Acad Sci U S A.

[26] Gorski A, Dabrowska K, Miedzybrodzki R, Weber-Dabrowska B, Lusiak-Szelachowska M, Jonczyk-Matysiak E (2017). Phages and immunomodulation. Future Microbiol.

[27] Shkoporov AN, Clooney AG, Sutton TDS, Ryan FJ, Daly KM, Nolan JA (2019). The human gut virome is highly diverse, stable, and individual specific. Cell Host Microbe.

[28] Yan Q, Wang Y, Chen X, Jin H, Wang G, Guan K (2021). Characterization of the gut DNA and RNA viromes in a cohort of Chinese residents and visiting Pakistanis. Virus Evol.

[29] Nakatsu G, Zhou H, Wu WKK, Wong SH, Coker OO, Dai Z (2018). Alterations in enteric virome are associated with colorectal cancer and survival outcomes. Gastroenterology.

[30] Chen F, Li S, Guo R, Song F, Zhang Y, Wang X (2023). Meta-analysis of fecal viromes demonstrates high diagnostic potential of the gut viral signatures for colorectal cancer and adenoma risk assessment. J Adv Res.

[31] Clooney AG, Sutton TDS, Shkoporov AN, Holohan RK, Daly KM, O'Regan O (2019). Whole-virome analysis sheds light on viral dark matter in inflammatory bowel disease. Cell Host Microbe.

[32] Zuo T, Lu XJ, Zhang Y, Cheung CP, Lam S, Zhang F (2019). Gut mucosal virome alterations in ulcerative colitis. Gut.

[33] Tian X, Li S, Wang C, Zhang Y, Feng X, Yan Q (2024). Gut virome-wide association analysis identifies cross-population viral signatures for inflammatory bowel disease. Microbiome.

[34] Jiang L, Lang S, Duan Y, Zhang X, Gao B, Chopyk J (2020). Intestinal virome in patients with alcoholic hepatitis. Hepatology.

[35] Lang S, Demir M, Martin A, Jiang L, Zhang X, Duan Y (2020). Intestinal virome signature associated with severity of nonalcoholic fatty liver disease. Gastroenterology.

[36] Tomofuji Y, Kishikawa T, Maeda Y, Ogawa K, Nii T, Okuno T (2022). Whole gut virome analysis of 476 Japanese revealed a link between phage and autoimmune disease. Ann Rheum Dis.

[37] Chen C, Yan Q, Yao X, Li S, Lv Q, Wang G (2023). Alterations of the gut virome in patients with systemic lupus erythematosus. Front Immunol.

[38] Guo R, Li S, Zhang Y, Zhang Y, Wang G, Ullah H (2022). Dysbiotic oral and gut viromes in untreated and treated rheumatoid arthritis patients. Microbiol Spectr.

[39] Mangalea MR, Paez-Espino D, Kieft K, Chatterjee A, Chriswell ME, Seifert JA (2021). Individuals at risk for rheumatoid arthritis harbor differential intestinal bacteriophage communities with distinct metabolic potential. Cell Host Microbe.

[40] Chen S, Zhou Y, Chen Y, Gu J (2018). fastp: an ultra-fast all-in-one FASTQ preprocessor. Bioinformatics.

[41] Langmead B, Salzberg SL (2012). Fast gapped-read alignment with Bowtie 2. Nat Methods.

[42] Li D, Liu CM, Luo R, Sadakane K, Lam TW (2015). MEGAHIT: an ultra-fast single-node solution for large and complex metagenomics assembly via succinct de Bruijn graph. Bioinformatics.

[43] Kieft K, Zhou Z, Anantharaman K (2020). VIBRANT: automated recovery, annotation and curation of microbial viruses, and evaluation of viral community function from genomic sequences. Microbiome.

[44] Nayfach S, Camargo AP, Schulz F, Eloe-Fadrosh E, Roux S, Kyrpides NC (2021). CheckV assesses the quality and completeness of metagenome-assembled viral genomes. Nat Biotechnol.

[45] Ren J, Song K, Deng C, Ahlgren NA, Fuhrman JA, Li Y (2020). Identifying viruses from metagenomic data using deep learning. Quantitative Biology.

[46] Li S, Guo R, Zhang Y, Li P, Chen F, Wang X (2022). A catalogue of 48,425 nonredundant viruses from oral metagenomes expands the horizon of the human oral virome. iScience.

[47] Manni M, Berkeley MR, Seppey M, Simao FA, Zdobnov EM (2021). BUSCO update: novel and streamlined workflows along with broader and deeper phylogenetic coverage for scoring of eukaryotic, prokaryotic, and viral genomes. Mol Biol Evol.

[48] Eddy SR (2011). Accelerated profile HMM searches. PLoS Comput Biol.

[49] Hyatt D, Chen G-L, LoCascio PF, Land ML, Larimer FW, Hauser LJ (2010). Prodigal: prokaryotic gene recognition and translation initiation site identification. BMC bioinformatics.

[50] Mihara T, Nishimura Y, Shimizu Y, Nishiyama H, Yoshikawa G, Uehara H (2016). Linking virus genomes with host taxonomy. Viruses.

[51] Guerin E, Shkoporov A, Stockdale SR, Clooney AG, Ryan FJ, Sutton TDS (2018). Biology and taxonomy of crAss-like bacteriophages, the most abundant virus in the human gut. Cell host & microbe.

[52] Benler S, Yutin N, Antipov D, Rayko M, Shmakov S, Gussow AB (2021). Thousands of previously unknown phages discovered in whole-community human gut metagenomes. Microbiome.

[53] Buchfink B, Xie C, Huson DH (2015). Fast and sensitive protein alignment using DIAMOND. Nature methods.

[54] Zhang P, Wang X, Li S, Cao X, Zou J, Fang Y (2023). Metagenome-wide association analysis implicates the gut bacteria and their functions in end-stage renal disease. Genome Biol.

[55] Skennerton C (2016). Minced—mining CRISPRs in environmental datasets. Github.

[56] Friedman J, Alm EJ (2012). Inferring correlation networks from genomic survey data. PLoS Comput Biol.

[57] Watts SC, Ritchie SC, Inouye M, Holt KE (2019). FastSpar: rapid and scalable correlation estimation for compositional data. Bioinformatics.

[58] Jie Z, Xia H, Zhong SL, Feng Q, Li S, Liang S (2017). The gut microbiome in atherosclerotic cardiovascular disease. Nat Commun.

[59] Tang S, Wu G, Liu Y, Xue B, Zhang S, Zhang W (2024). Guild-level signature of gut microbiome for diabetic kidney disease. mBio.

[60] Yu J, Feng Q, Wong SH, Zhang D, Liang QY, Qin Y (2017). Metagenomic analysis of faecal microbiome as a tool towards targeted non-invasive biomarkers for colorectal cancer. Gut.

[61] Yan Q, Gu Y, Li X, Yang W, Jia L, Chen C (2017). Alterations of the gut microbiome in hypertension. Front Cell Infect Microbiol.

[62] He Q, Gao Y, Jie Z, Yu X, Laursen JM, Xiao L (2017). Two distinct metacommunities characterize the gut microbiota in Crohn's disease patients. Gigascience.

[63] Qin N, Yang F, Li A, Prifti E, Chen Y, Shao L (2014). Alterations of the human gut microbiome in liver cirrhosis. Nature.

[64] Liu R, Hong J, Xu X, Feng Q, Zhang D, Gu Y (2017). Gut microbiome and serum metabolome alterations in obesity and after weight-loss intervention. Nat Med.

[65] Zhang X, Zhang D, Jia H, Feng Q, Wang D, Liang D (2015). The oral and gut microbiomes are perturbed in rheumatoid arthritis and partly normalized after treatment. Nat Med.

[66] Qin J, Li Y, Cai Z, Li S, Zhu J, Zhang F (2012). A metagenome-wide association study of gut microbiota in type 2 diabetes. Nature.

[67] Zeller G, Tap J, Voigt AY, Sunagawa S, Kultima JR, Costea PI (2014). Potential of fecal microbiota for early-stage detection of colorectal cancer. Mol Syst Biol.

[68] Feng Q, Liang S, Jia H, Stadlmayr A, Tang L, Lan Z (2015). Gut microbiome development along the colorectal adenoma-carcinoma sequence. Nat Commun.

[69] Thomas AM, Manghi P, Asnicar F, Pasolli E, Armanini F, Zolfo M (2019). Metagenomic analysis of colorectal cancer datasets identifies cross-cohort microbial diagnostic signatures and a link with choline degradation. Nat Med.

[70] Wirbel J, Pyl PT, Kartal E, Zych K, Kashani A, Milanese A (2019). Meta-analysis of fecal metagenomes reveals global microbial signatures that are specific for colorectal cancer. Nat Med.

[71] Vogtmann E, Hua X, Zeller G, Sunagawa S, Voigt AY, Hercog R (2016). Colorectal cancer and the human gut microbiome: reproducibility with whole-genome shotgun sequencing. PLoS One.

[72] Yachida S, Mizutani S, Shiroma H, Shiba S, Nakajima T, Sakamoto T (2019). Metagenomic and metabolomic analyses reveal distinct stage-specific phenotypes of the gut microbiota in colorectal cancer. Nat Med.

[73] Gregory AC, Zayed AA, Conceicao-Neto N, Temperton B, Bolduc B, Alberti A (2019). Marine DNA viral macro- and microdiversity from pole to pole. Cell.

[74] Roux S, Adriaenssens EM, Dutilh BE, Koonin EV, Kropinski AM, Krupovic M (2019). Minimum information about an uncultivated virus genome (MIUViG). Nat Biotechnol.

[75] Yutin N, Benler S, Shmakov SA, Wolf YI, Tolstoy I, Rayko M (2021). Analysis of metagenome-assembled viral genomes from the human gut reveals diverse putative CrAss-like phages with unique genomic features. Nat Commun.

[76] Siranosian BA, Tamburini FB, Sherlock G, Bhatt AS (2020). Acquisition, transmission and strain diversity of human gut-colonizing crAss-like phages. Nat Commun.

[77] Dronavalli S, Duka I, Bakris GL (2008). The pathogenesis of diabetic nephropathy. Nat Clin Pract Endocrinol Metab.

[78] Manrique P, Dills M, Young MJ (2017). The human gut phage community and its implications for health and disease. Viruses.

[79] Bai L, Xie T, Hu Q, Deng C, Zheng R, Chen W (2015). Genome-wide comparison of ferritin family from archaea, bacteria, eukarya, and viruses: its distribution, characteristic motif, and phylogenetic relationship. Naturwissenschaften.

[80] Shkoporov AN, Khokhlova EV, Fitzgerald CB, Stockdale SR, Draper LA, Ross RP (2018). PhiCrAss001 represents the most abundant bacteriophage family in the human gut and infects Bacteroides intestinalis. Nat Commun.

[81] Ma Y, You X, Mai G, Tokuyasu T, Liu C (2018). A human gut phage catalog correlates the gut phageome with type 2 diabetes. Microbiome.

[82] Hryckowian AJ, Merrill BD, Porter NT, Van Treuren W, Nelson EJ, Garlena RA (2020). Bacteroides thetaiotaomicron-infecting bacteriophage isolates inform sequence-based host range predictions. Cell Host Microbe.

[83] Zhao J, Ning X, Liu B, Dong R, Bai M, Sun S (2021). Specific alterations in gut microbiota in patients with chronic kidney disease: an updated systematic review. Ren Fail.

[84] Machiels K, Joossens M, Sabino J, De Preter V, Arijs I, Eeckhaut V (2014). A decrease of the butyrate-producing species Roseburia hominis and Faecalibacterium prausnitzii defines dysbiosis in patients with ulcerative colitis. Gut.

[85] Zhou L, Zhang M, Wang Y, Dorfman RG, Liu H, Yu T (2018). Faecalibacterium prausnitzii produces butyrate to maintain Th17/Treg balance and to ameliorate colorectal colitis by inhibiting histone deacetylase 1. Inflamm Bowel Dis.

[86] Cornuault JK, Petit MA, Mariadassou M, Benevides L, Moncaut E, Langella P (2018). Phages infecting Faecalibacterium prausnitzii belong to novel viral genera that help to decipher intestinal viromes. Microbiome.

[87] Duncan SH, Hold GL, Barcenilla A, Stewart CS, Flint HJ (2002). Roseburia intestinalis sp. nov, a novel saccharolytic, butyrate-producing bacterium from human faeces. Int J Syst Evol Microbiol.

[88] Koh A, De Vadder F, Kovatcheva-Datchary P, Backhed F (2016). From dietary fiber to host physiology: short-chain fatty acids as key bacterial metabolites. Cell.

[89] Thompson LR, Zeng Q, Kelly L, Huang KH, Singer AU, Stubbe J (2011). Phage auxiliary metabolic genes and the redirection of cyanobacterial host carbon metabolism. Proc Natl Acad Sci U S A.

[90] Kieft K, Breister AM, Huss P, Linz AM, Zanetakos E, Zhou Z (2021). Virus-associated organosulfur metabolism in human and environmental systems. Cell Rep.

[91] Kieft K, Zhou Z, Anderson RE, Buchan A, Campbell BJ, Hallam SJ (2021). Ecology of inorganic sulfur auxiliary metabolism in widespread bacteriophages. Nat Commun.

[92] Militello KT, Simon RD, Qureshi M, Maines R, VanHorne ML, Hennick SM (2012). Conservation of Dcm-mediated cytosine DNA methylation in Escherichia coli. FEMS Microbiol Lett.

[93] Carding SR, Davis N, Hoyles L (2017). Review article: the human intestinal virome in health and disease. Aliment Pharmacol Ther.

[94] Nayfach S, Paez-Espino D, Call L, Low SJ, Sberro H, Ivanova NN (2021). Metagenomic compendium of 189,680 DNA viruses from the human gut microbiome. Nat Microbiol.

[95] Huang L, Guo R, Li S, Wu X, Zhang Y, Guo S (2024). A multi-kingdom collection of 33,804 reference genomes for the human vaginal microbiome. Nat Microbiol.

[96] Wang G, Li S, Yan Q, Guo R, Zhang Y, Chen F (2023). Optimization and evaluation of viral metagenomic amplification and sequencing procedures toward a genome-level resolution of the human fecal DNA virome. J Adv Res.

[97] Arumugam M, Raes J, Pelletier E, Le Paslier D, Yamada T, Mende DR (2011). Enterotypes of the human gut microbiome. Nature.

[98] Human Microbiome Project C (2012). A framework for human microbiome research. Nature.

[99] Zuo T, Sun Y, Wan Y, Yeoh YK, Zhang F, Cheung CP (2020). Human-gut-DNA virome variations across geography, ethnicity, and urbanization. Cell Host Microbe.

[100] Brussow H, Desiere F (2001). Comparative phage genomics and the evolution of Siphoviridae: insights from dairy phages. Mol Microbiol.

[101] Doore SM, Fane BA (2016). The microviridae: diversity, assembly, and experimental evolution. Virology.

[102] Shah SA, Deng L, Thorsen J, Pedersen AG, Dion MB, Castro-Mejía JL (2023). Expanding known viral diversity in the healthy infant gut. Nat Microbiol.

[103] Curtis MM, Hu Z, Klimko C, Narayanan S, Deberardinis R, Sperandio V (2014). The gut commensal Bacteroides thetaiotaomicron exacerbates enteric infection through modification of the metabolic landscape. Cell Host Microbe.

[104] Hall AB, Yassour M, Sauk J, Garner A, Jiang X, Arthur T (2017). A novel Ruminococcus gnavus clade enriched in inflammatory bowel disease patients. Genome Med.

[105] Henke MT, Brown EM, Cassilly CD, Vlamakis H, Xavier RJ, Clardy J (2021). Capsular polysaccharide correlates with immune response to the human gut microbe Ruminococcus gnavus. Proc Natl Acad Sci U S A.

[106] Le Chatelier E, Nielsen T, Qin J, Prifti E, Hildebrand F, Falony G (2013). Richness of human gut microbiome correlates with metabolic markers. Nature.

[107] Woting A, Pfeiffer N, Loh G, Klaus S, Blaut M (2014). Clostridium ramosum promotes high-fat diet-induced obesity in gnotobiotic mouse models. mBio.

[108] Dehoux P, Marvaud JC, Abouelleil A, Earl AM, Lambert T, Dauga C (2016). Comparative genomics of Clostridium bolteae and Clostridium clostridioforme reveals species-specific genomic properties and numerous putative antibiotic resistance determinants. BMC Genomics.

[109] Gupta A, Dhakan DB, Maji A, Saxena R, P KV, Mahajan S (2019). Association of Flavonifractor plautii, a flavonoid-degrading bacterium, with the gut microbiome of colorectal cancer patients in India. mSystems.

[110] Liu X, Mao B, Gu J, Wu J, Cui S, Wang G (2021). Blautia-a new functional genus with potential probiotic properties?. Gut Microbes.

[111] Hurwitz BL, Hallam SJ, Sullivan MB (2013). Metabolic reprogramming by viruses in the sunlit and dark ocean. Genome Biol.

[112] Yamaguchi H, Furuhata K, Fukushima T, Yamamoto H, Sekiguchi J (2004). Characterization of a new Bacillus subtilis peptidoglycan hydrolase gene, yvcE (named cwlO), and the enzymatic properties of its encoded protein. J Biosci Bioeng.

[113] Hashimoto M, Ooiwa S, Sekiguchi J (2012). Synthetic lethality of the lytE cwlO genotype in Bacillus subtilis is caused by lack of D,L-endopeptidase activity at the lateral cell wall. J Bacteriol.

[114] Rodriguez-Rubio L, Martinez B, Donovan DM, Rodriguez A, Garcia P (2013). Bacteriophage virion-associated peptidoglycan hydrolases: potential new enzybiotics. Crit Rev Microbiol.

[115] Silpe JE, Bassler BL (2019). A host-produced quorum-sensing autoinducer controls a phage lysis-lysogeny decision. Cell.

[116] Carroll-Portillo A, Lin HC (2019). Bacteriophage and the innate immune system: access and signaling. Microorganisms.

[117] Vollmer W, Joris B, Charlier P, Foster S (2008). Bacterial peptidoglycan (murein) hydrolases. FEMS Microbiol Rev.

[118] Lee JY, Li Z, Miller ES (2017). Vibrio phage KVP40 encodes a functional NAD^+^ salvage pathway. J Bacteriol.

[119] Minhas PS, Liu L, Moon PK, Joshi AU, Dove C, Mhatre S (2019). Macrophage de novo NAD^+^ synthesis specifies immune function in aging and inflammation. Nat Immunol.

[120] Skliros D, Kalatzis PG, Katharios P, Flemetakis E (2016). Comparative functional genomic analysis of two vibrio phages reveals complex metabolic interactions with the host cell. Front Microbiol.

[121] Poyan Mehr A, Tran MT, Ralto KM, Leaf DE, Washco V, Messmer J (2018). De novo NAD^+^ biosynthetic impairment in acute kidney injury in humans. Nature medicine.

[122] Chanvillard L, Tammaro A, Sorrentino V (2022). NAD^+^ metabolism and interventions in premature renal aging and chronic kidney disease. Cells.

[123] Bignon Y, Rinaldi A, Nadour Z, Poindessous V, Nemazanyy I, Lenoir O (2022). Cell stress response impairs de novo NAD^+^ biosynthesis in the kidney. JCI insight.

[124] Duvallet C, Gibbons SM, Gurry T, Irizarry RA, Alm EJ (2017). Meta-analysis of gut microbiome studies identifies disease-specific and shared responses. Nat Commun.

[125] Jackson MA, Verdi S, Maxan ME, Shin CM, Zierer J, Bowyer RCE (2018). Gut microbiota associations with common diseases and prescription medications in a population-based cohort. Nat Commun.

[126] Yan Q, Li S, Yan Q, Huo X, Wang C, Wang X (2024). A genomic compendium of cultivated human gut fungi characterizes the gut mycobiome and its relevance to common diseases. Cell.

[127] Fan Y, Pedersen O (2021). Gut microbiota in human metabolic health and disease. Nat Rev Microbiol.

[128] Foxman EF, Iwasaki A (2011). Genome-virome interactions: examining the role of common viral infections in complex disease. Nat Rev Microbiol.

[129] Adiliaghdam F, Jeffrey KL (2020). Illuminating the human virome in health and disease. Genome Med.

[130] Wu IW, Gao SS, Chou HC, Yang HY, Chang LC, Kuo YL (2020). Integrative metagenomic and metabolomic analyses reveal severity-specific signatures of gut microbiota in chronic kidney disease. Theranostics.

