# Supplementary material for: A metagenome-wide study of the gut virome in chronic kidney disease
Source: Theranostics. 2025 Jan 2;15(5):1642–61. doi: 10.7150/thno.101601 (PMC11780533; doi:10.7150/thno.101601)
Supplement: Supplementary file 2 — Supplementary Additional file 2: Figure S1. Construction of the vOTU catalogue and comparison with other databases. Figure S2. Host assignment of the vOTU catalogue. Figure S3. Composition of the samples with the highest proportion of viruses. Figure S4. Comparison of alpha diversity indexes and family-level compositions between CKD patients and healthy controls. Figure S5. Receiver operating characteristic (ROC) analysis of the classification of CKD status. Figure S6. Comparison of gut virome among CKD patients at different clinical stages. Figure S7. Comparison of gut virome among CKD patients with different protopathies. Figure S8. Identification of CKD-associated vOTUs. Figure S9. Functional comparison between the bacterium-dependent and bacterium-independent vOTUs. Figure S10. Comparison of host-phage pairs and statistical relationships. Figure S11. Virus-bacterium networks for Bacteroides, Prevotella, and Faecalibacterium spp. and their related viruses. Figure S12. Genetic distances between Bacteroides-connected vOTUs and existing B. thetaiotaomicron-infecting phages. Figure S13. Correlations between the Eggerthella/Fusobacterium-related vOTUs and the serum uremic toxin concentrations of individuals. Figure S14. Comparison of the functional composition between CKD-enriched and HC-enriched vOTUs. Figure S15. Enzymes that involved in NAD+ de novo biosynthesis and salvage. Figure S16. Distribution of 25 NAD+ biosynthesis-associated enzymes in the faecal metagenomes of all samples. Figure S17. Detection rate of 4,649 CKD-associated vOTUs in 9 cohorts. Figure S18. Alterations of CKD-associated vOTUs in 6 studies of CRC faecal microbiome. [file thnov15p1642s2.pdf]

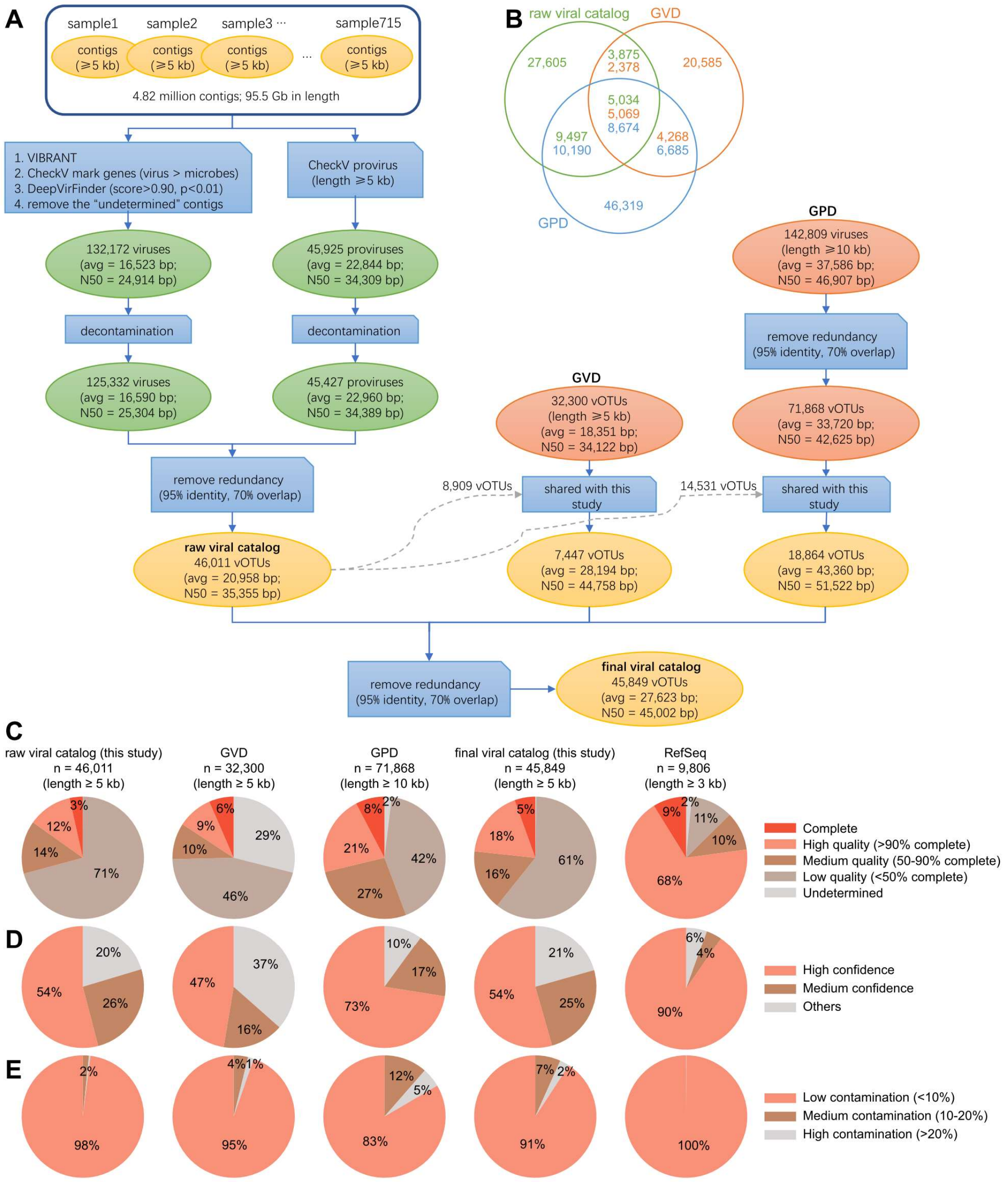

Supplementary figure 1. Construction of the vOTU catalogue and comparison with other databases. (A) Workflow for the construction of a non-redundant vOTU catalogue based on 715 faecal metagenomes in this study. (B) Venn plot shows the overlap of the virus among the GVD, GPD, and raw viral catalogue of this study. (C-E) CheckV estimation of the completeness (C), confidence (D), and contamination (E) of five viral catalogues.

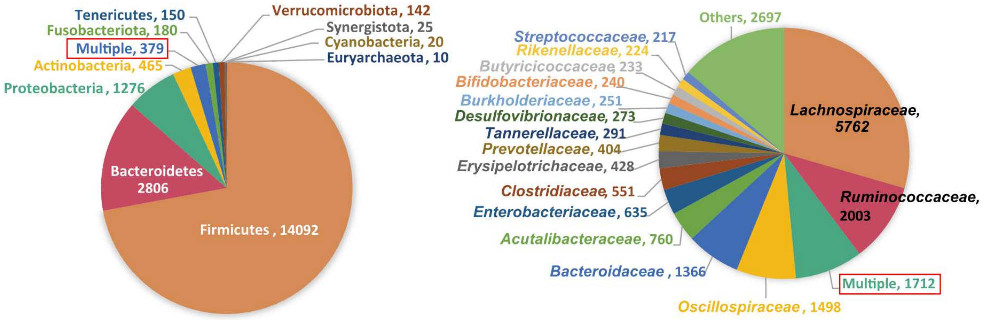

Supplementary figure 2. Host assignment of the vOTU catalogue.

Pie plots show the proportions of prokaryotic hosts at the phylum (left panel) and family (right panel) levels for 19,545 vOTUs. Red boxes label the number of vOTUs that have the hosts belonged to >2 bacterial phyla or families.

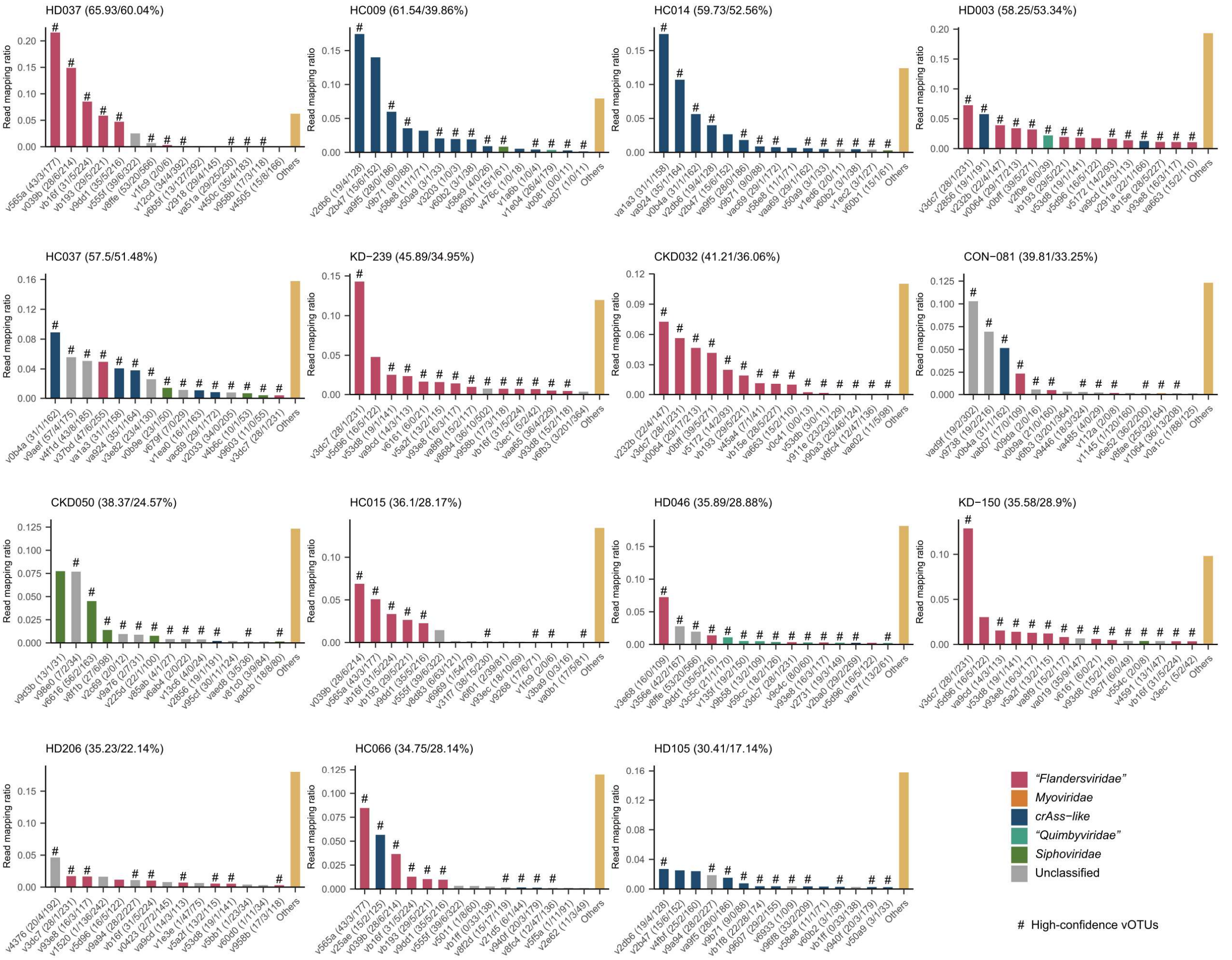

Supplementary figure 3. Composition of the samples with the highest proportion of viruses.
15 samples with >30% of reads mapping into the vOTU catalogue are shown. For each sample, the bar plot shows the proportion of reads for each vOTU, and the colors of bars represent the family-level taxonomic assignment of the vOTUs. The numbers behind the sample ID indicate the accumulated proportion of all vOTUs versus the accumulated proportion of high-confidence vOTUs. The numbers behind the vOTU ID indicate the numbers of viral genes (identified by CheckV), microbial genes, and total predicted genes.

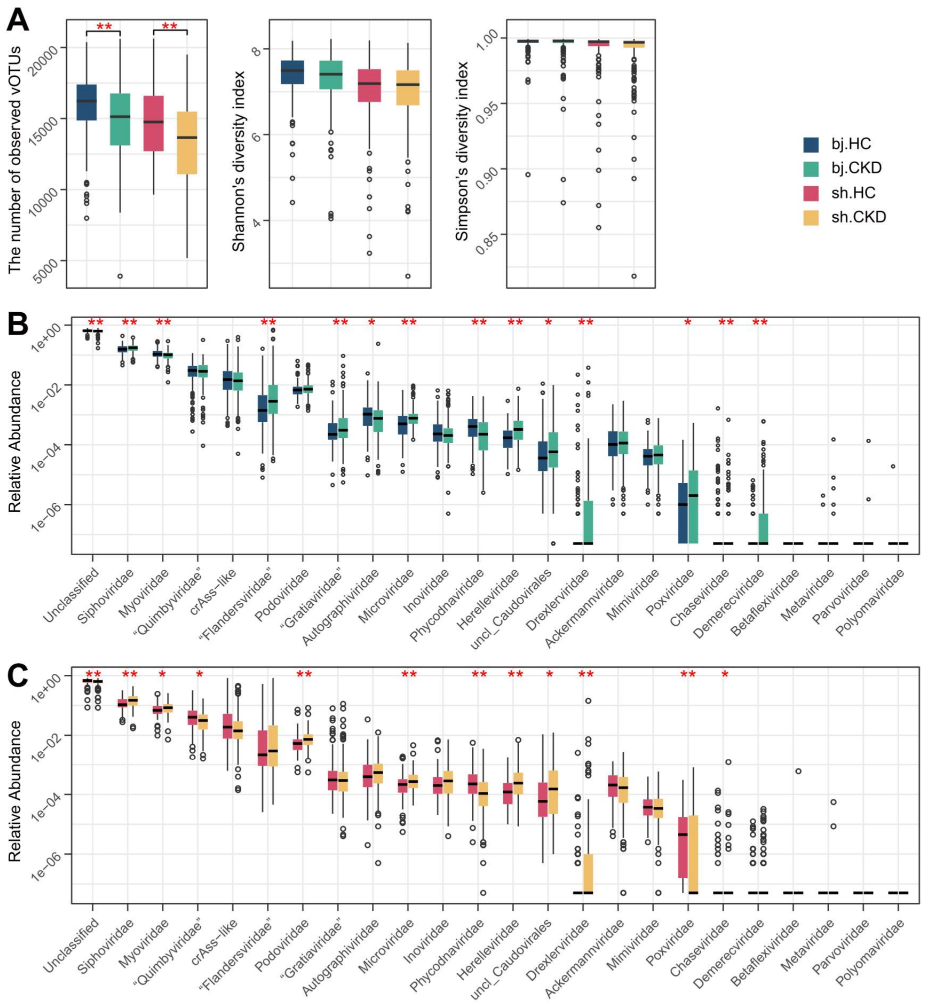

Supplementary figure 4. Comparison of alpha diversity indexes and family-level compositions between CKD patients and healthy controls.

(A) Boxplots show the distribution of 3 within-sample (alpha) diversity indexes, including the number of observed vOTUs, Shannon index, and Simpson index, among groups. Boxes represent the interquartile range between the first and third quartiles and the median (internal line). Whiskers denote the lowest and highest values within 1.5 times the range of the first and third quartiles, respectively; dots represent outlier samples beyond the whiskers. (B-C) Boxplots show the composition of gut virome at the family level, for the faecal metagenomes of the Beijing (B) and Shanghai (C) cohorts. Wilcoxon rank-sum test: *, q<0.05; **, q<0.01; ***, q<0.001.

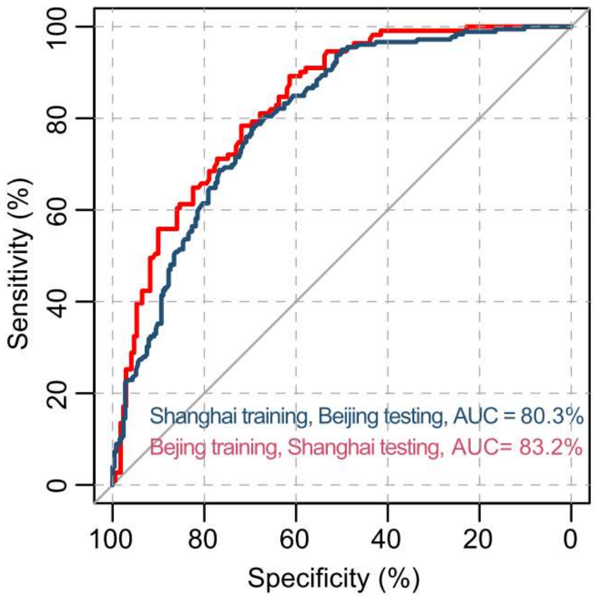

Supplementary figure 5. Receiver operating characteristic (ROC) analysis of the classification of CKD status.

The performance of random forest models trained from the gut viral profiles of Shanghai individuals for classifying the Beijing individuals (blue line) and from the gut viral profiles of Beijing individuals for classifying the Shanghai individuals (red line) are shown.

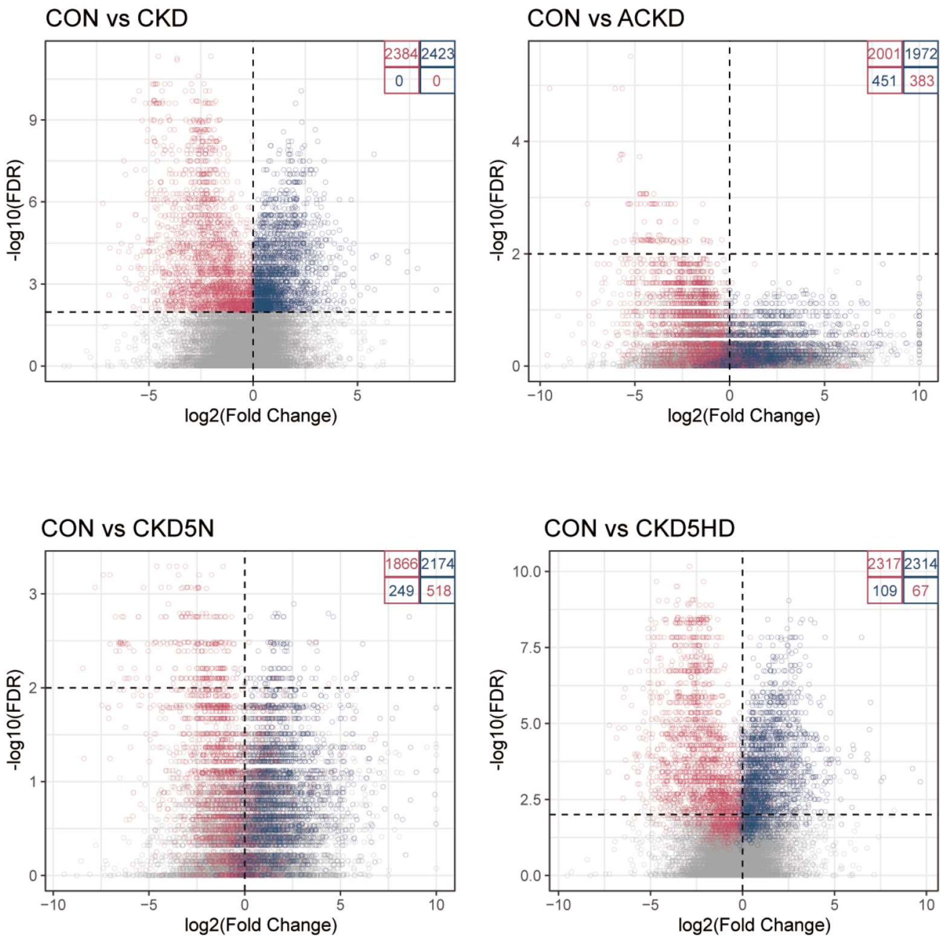

Supplementary figure 6. Comparison of gut virome among CKD patients at different clinical stages. Volcano plots show the fold change vs. q-values for pairwise comparisons. The X-axis shows the fold change ratio (log2 transformed) of a vOTU abundance in CKD patients compared with healthy controls (fold change <0, CKD-enriched; fold change >0, HC-enriched). and the Y-axis shows the q-value (-log10 transformed) of a vOTU species, and the dotted line indicates q-value <0.01. The upper left panel shows the comparison between all CKD patients and healthy controls. For this comparison, 2,384 and 2,423 vOTUs are identified with significant enrichment in relative abundance of CKD patients (red points) and healthy controls (blue points), respectively. The other three panels show the comparisons of ACKD patients (upper right), CKD5N patients (bottom left), and CKD5HD patients (bottom right) versus healthy controls. For these comparisons, the 2,384 CKD-enriched and 2,423 HC-enriched vOTUs are labeled by red and blue points, respectively.

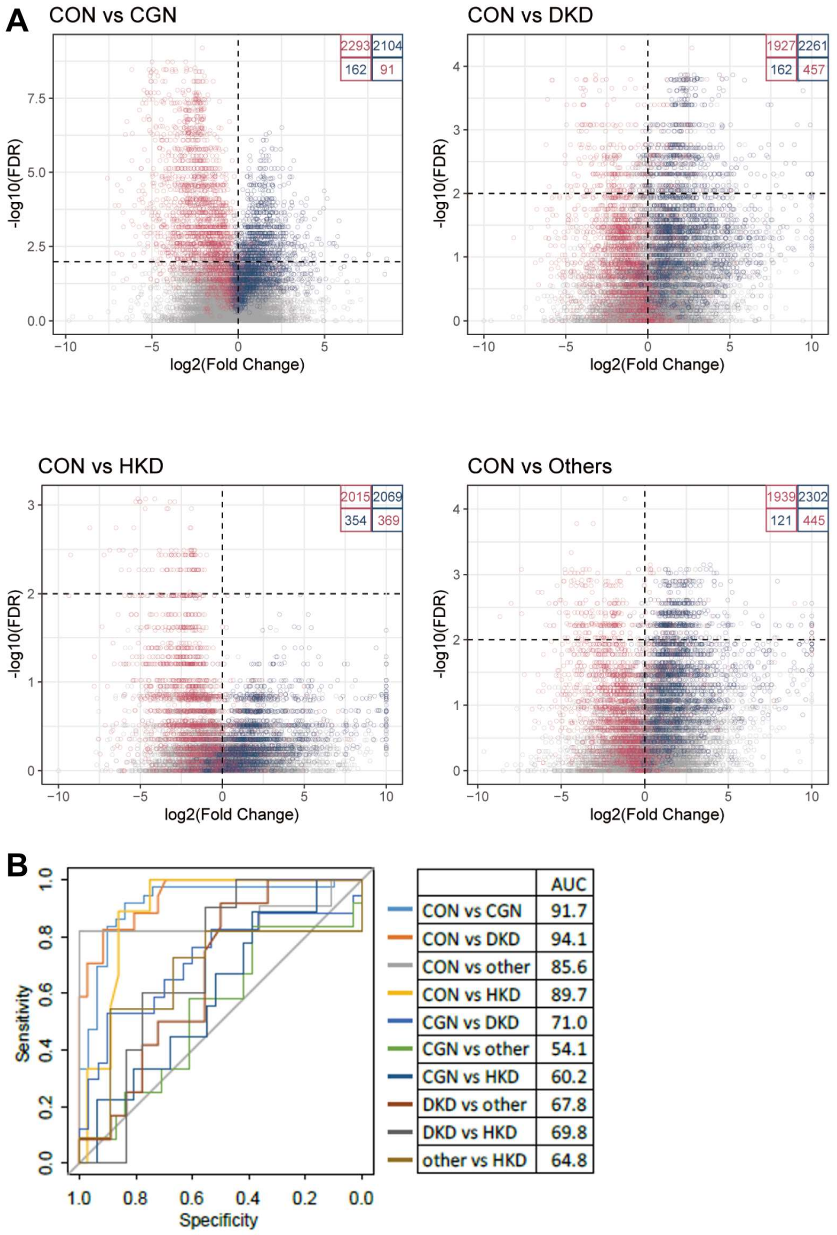

Supplementary figure 7. Comparison of gut virome among CKD patients with different protopathies. (A) Volcano plots show the fold change vs. q-values for pairwise comparisons. The X-axis shows the fold change ratio (log2 transformed) of a vOTU abundance in CKD patients compared with healthy controls (fold change <0, CKD-enriched; fold change >0, HC-enriched). and the Y-axis shows the q-value (-log10 transformed) of a vOTU species, and the dotted line indicates q-value <0.01. The panels show the comparisons of CGN patients (upper left), DKD patients (upper right), HKD patients (bottom left), and others (bottom right) versus healthy controls. For these comparisons, the 2,384 CKD-enriched and 2,423 HC-enriched vOTUs (explained in Supplementary figure 6) are labeled by red and blue points, respectively. (B) Receiver operating characteristic (ROC) analysis of the classification of different groups. For each random forest model, 70% of the samples were randomly selected as the training set, and the remaining 30% of the samples were used as the testing set. The classification performance of the model was assessed by the area under the ROC curve (AUC).

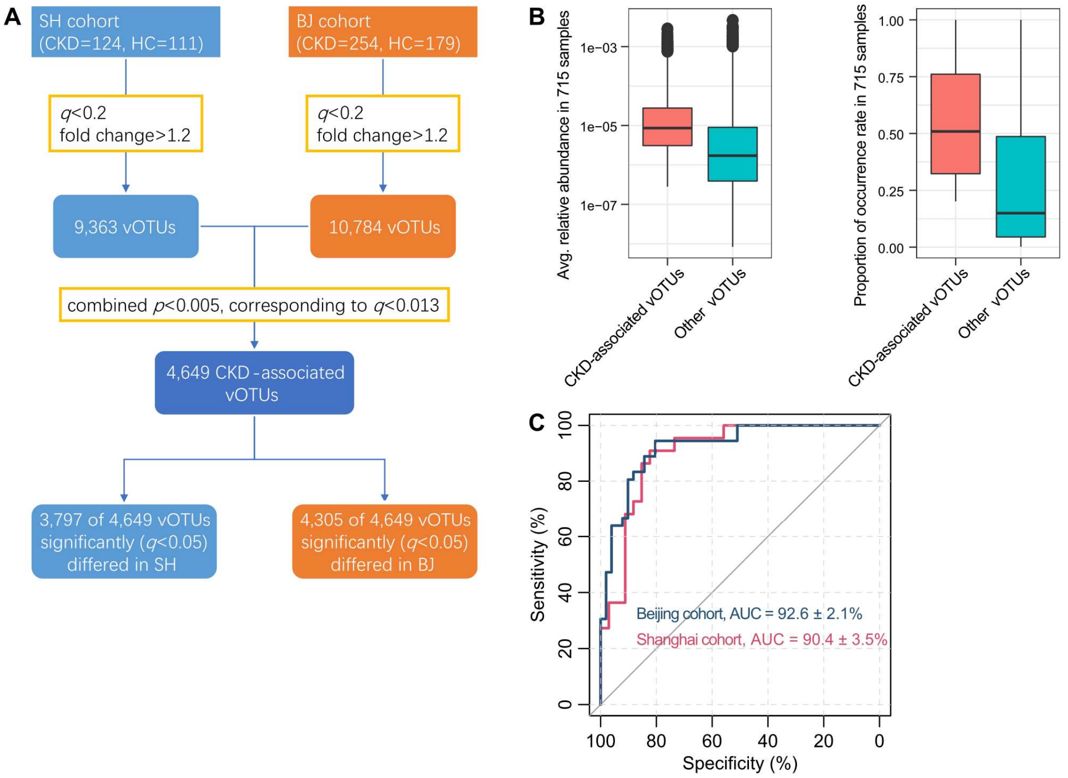

Supplementary figure 8. Identification of CKD-associated vOTUs.

(A) Overall schematic to identify the CKD-associated vOTUs from two independent cohorts. (B) The distribution of average relative abundance (left panel) and proportion of occurrence rate (right panel) of 4,649 CKD-associated vOTUs versus other vOTUs, in 715 faecal metagenomes. (C) Receiver operating characteristic (ROC) analysis of the classification of CKD status based on the abundance 4,649 CKD-associated vOTUs. For each cohort, 70% of the samples were randomly selected as the training set for the random forest model, and the remaining 30% of the samples were used as the testing set. The classification performance of the model was assessed by the area under the ROC curve (AUC). The AUC values and 95% confidence intervals (CIs) are shown.

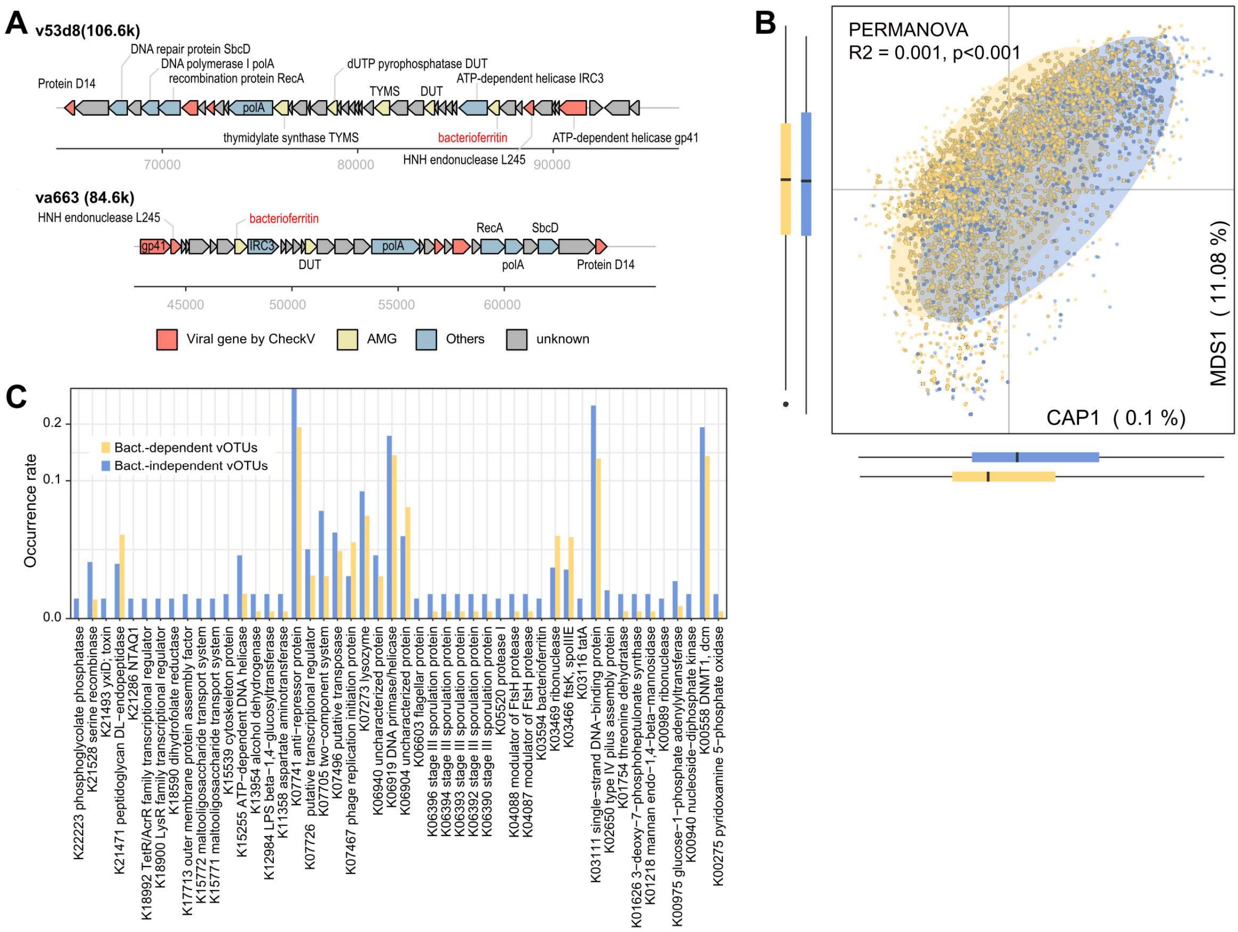

Supplementary figure 9. Functional comparison between the bacterium-dependent and bacterium-independent vOTUs.

(A) Two vOTUs belonged to “Flandersviridae” encode a bacterioferritin gene. (B) Distance-based redundancy analysis (dbRDA) of the Bray-Curtis distance for the functional composition of all analyzed vOTUs. Display is based on vOTU scores on the primary constrained axis (CAP1) and primary multidimensional scaling (MDS1). Ellipsoids represent a 95% confidence interval surrounding each group. (C) Barplot shows the occurrence rate of the KEGG orthologs that differed in frequency between the bacterium-dependent and bacterium-independent vOTUs.

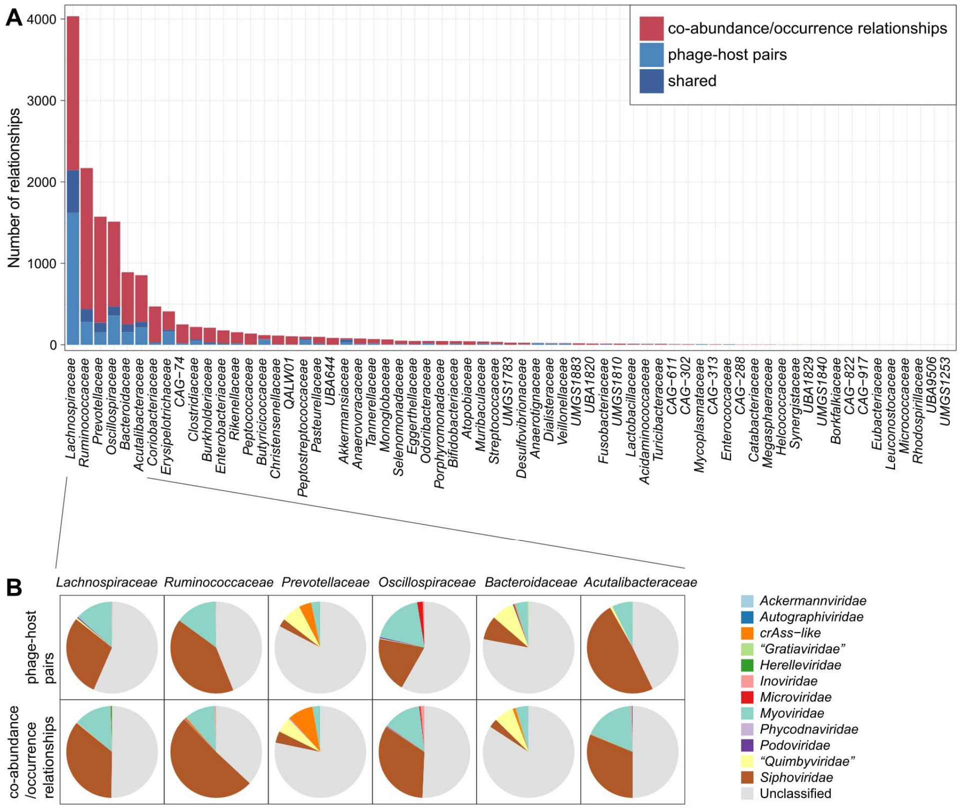

Supplementary figure 10. Comparison of host-phage pairs and statistical relationships.

(A) Barplot showing the number of relationships between vOTUs and bacteria. Bacteria are grouped by their taxonomic assignment at the family level. (B) Pie plots showing the proportion of viruses existing host-phage pairs (upper pies) and co-abundance/occurrence relationships (bottom pies) within different bacterial families. The high similarity of viral composition between two types of relationships suggested that they may derive from the same biological pattern.

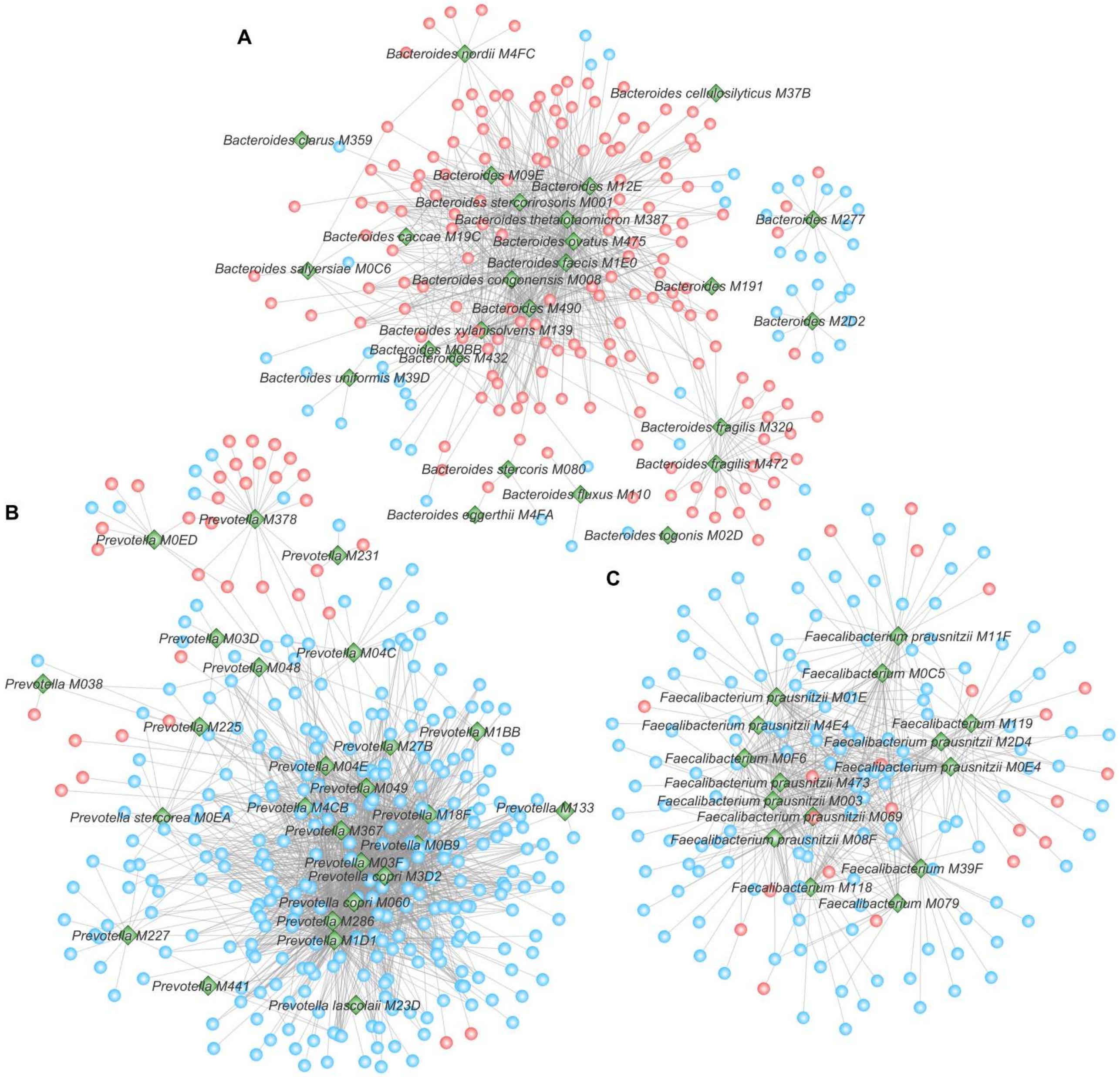

Supplementary figure 11. Virus-bacterium networks for *Bacteroides* (A), *Prevotella* (B), and *Faecalibacterium* spp. (C) and their related viruses.

Red and blue nodes represent the vOTUs that enriched in CKD patients and healthy controls, respectively. Green nodes represent the bacterial species.

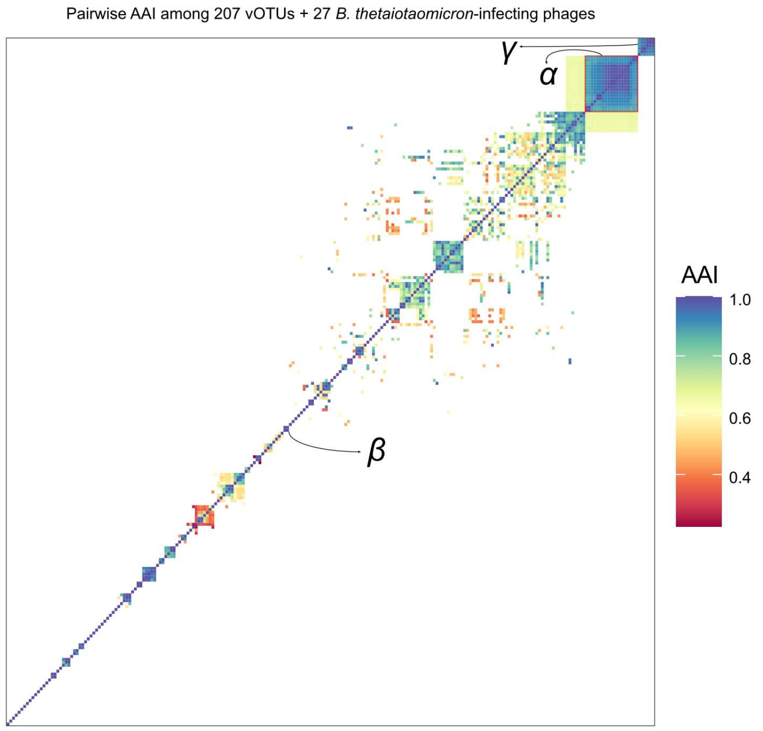

Supplementary figure 12. Genetic distances between *Bacteroides*-connected vOTUs and existing *B. thetaiotaomicron*-infecting phages.

Heatmap showing the pairwise average amino acid identity (AAI) among 207 vOTUs and 27 *B. thetaiotaomicron*-infecting phages. Three subclades of *B. thetaiotaomicron*-infecting phages are labeled.

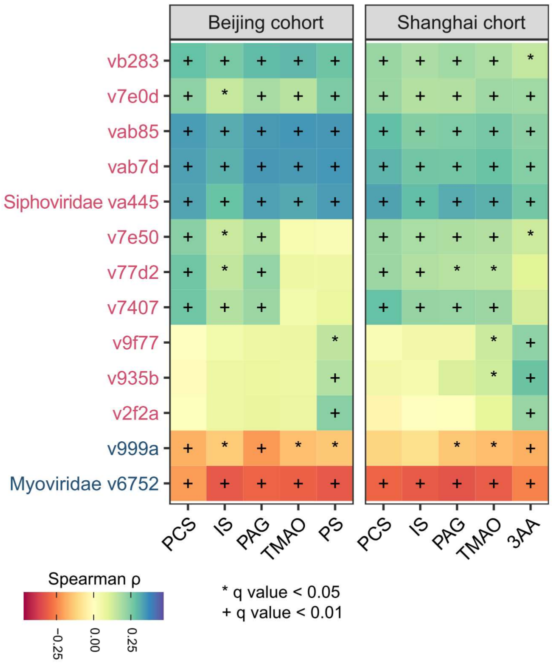

Supplementary figure 13. Correlations between the *Eggerthella*/ *Fusobacterium*-related vOTUs and the serum uremic toxin concentrations of individuals.

Heatmap shows the Spearman correlation coefficients between the 13 CKD-associated vOTUs that related to *Eggerthella* spp. and *Fusobacterium* spp. and the serum uremic toxin concentrations of individuals. PCS, *p*-cresol sulfate; IS, indoxyl sulfate; PAG, phenylacetylglutamine; TMAO, trimethylamine N- oxide; PS, phenyl sulfate; 3AA, indole-3-acetic acid.

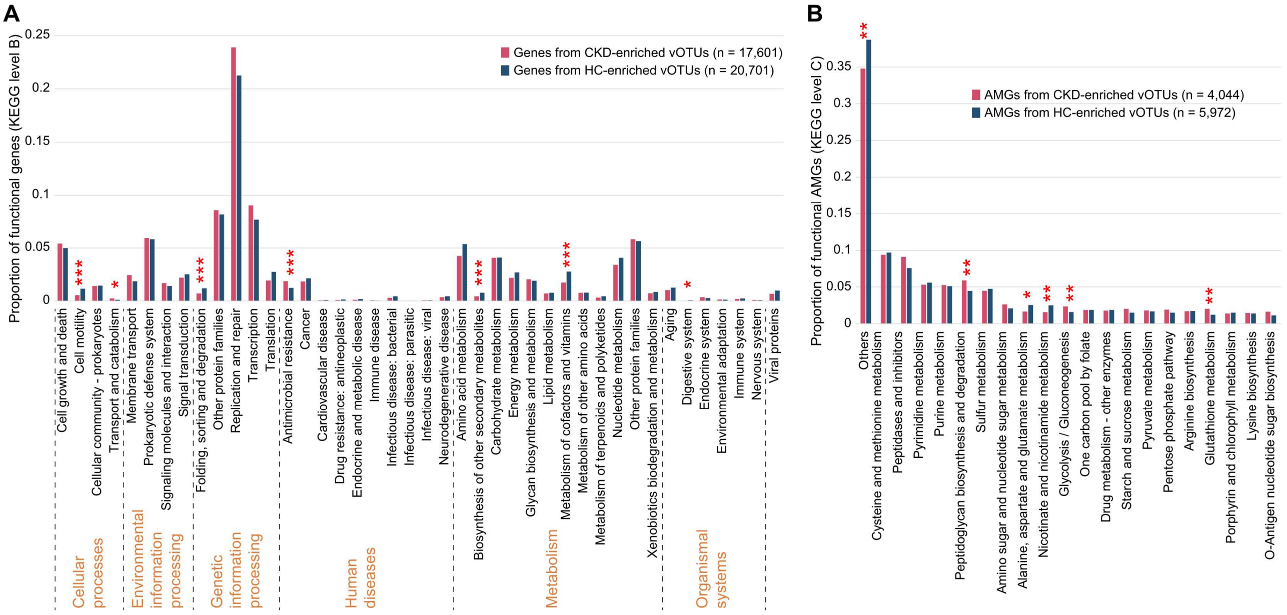

Supplementary figure 14. Comparison of the functional composition between CKD-enriched and HC-enriched vOTUs.

(A) Barplot shows the distribution of functional composition categorized at the KEGG pathway level B. (B) Barplot shows the composition of viral AMGs. Fisher's exact test: * $q<0.05$; ** $q<0.01$; *** $q<0.001$.

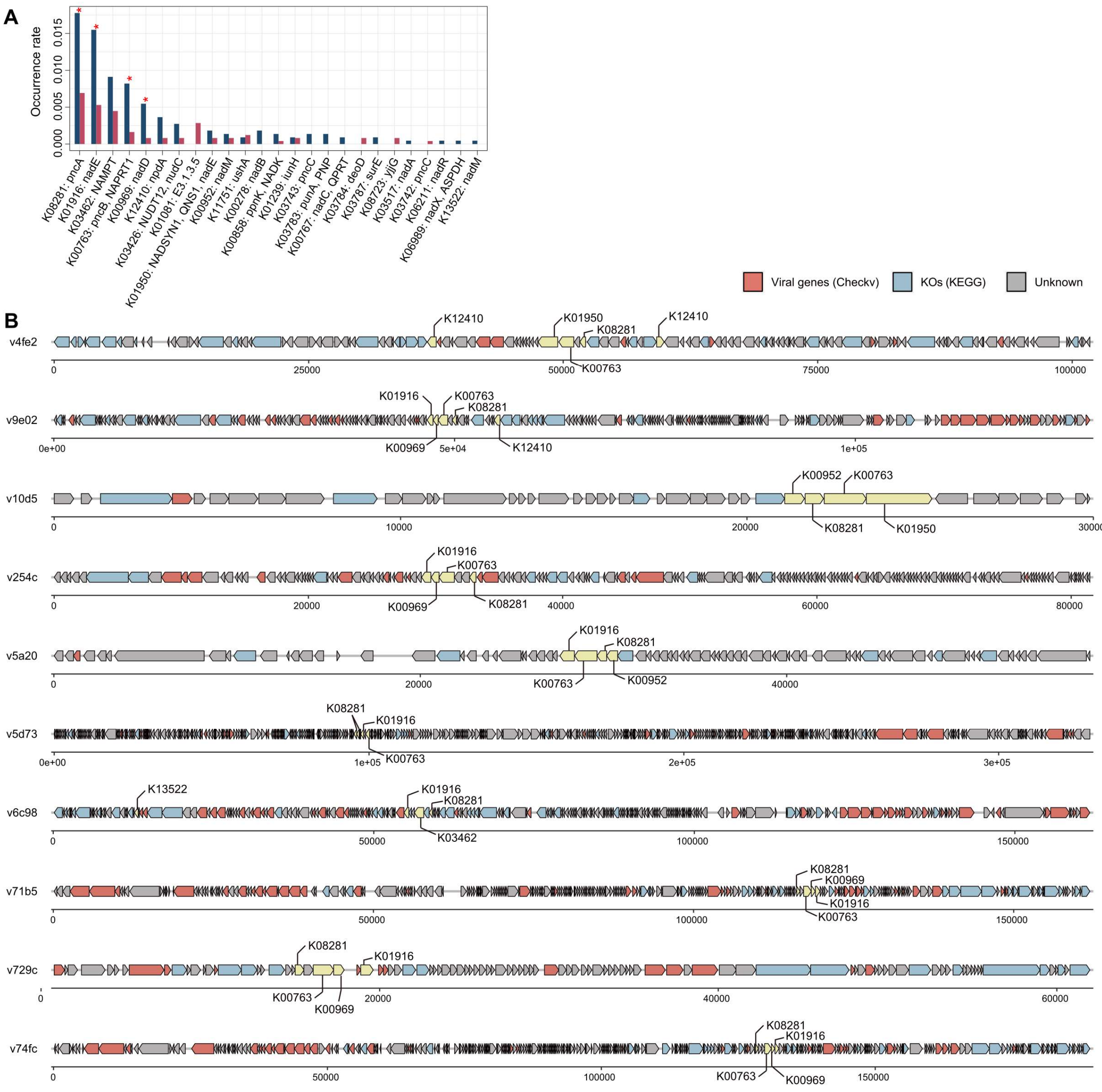

Supplementary figure 15. Enzymes that involved in NAD+ *de novo* biosynthesis and salvage.

(A) Barplot shows the occurrent rate of 25 enzymes in the CKD-enriched (red) and HC-enriched (blue) vOTUs. Fisher's exact test: *, q<0.05. (B) Representative alignment of 10 viral contigs that encoded more than 4 enzymes involving NAD+ biosynthesis. The viral genes of the contigs are identified by CheckV.

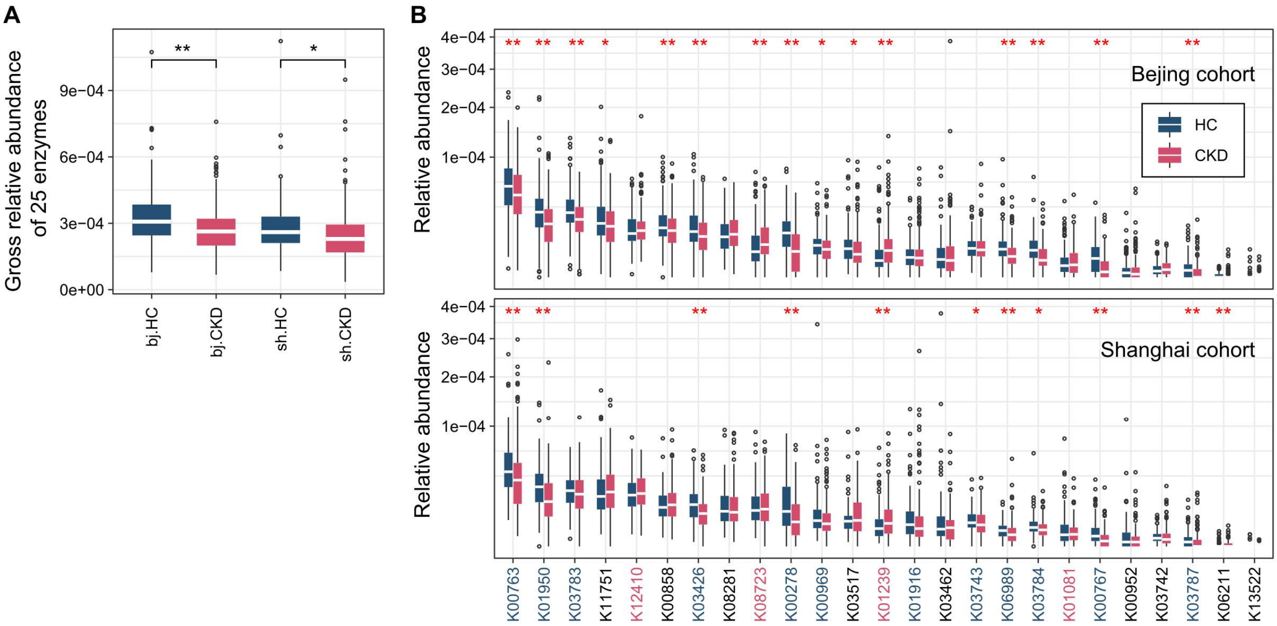

Supplementary figure 16. Distribution of 25 NAD+ biosynthesis-associated enzymes in the faecal metagenomes of all samples.

(A) Boxplot shows the gross relative abundance of 25 enzymes in the faecal metagenomes. (B) Boxplots show the relative abundance of each of 25 enzymes in the faecal metagenomes of Beijing (upper panel) and Shanghai (bottom panel) cohorts. Boxes represent the interquartile range between the first and third quartiles and the median (internal line). Whiskers denote the lowest and highest values within 1.5 times the range of the first and third quartiles, respectively; dots represent outlier samples beyond the whiskers. Student's t-test: *, $q<0.05$; **, $q<0.01$; ***, $q<0.001$.

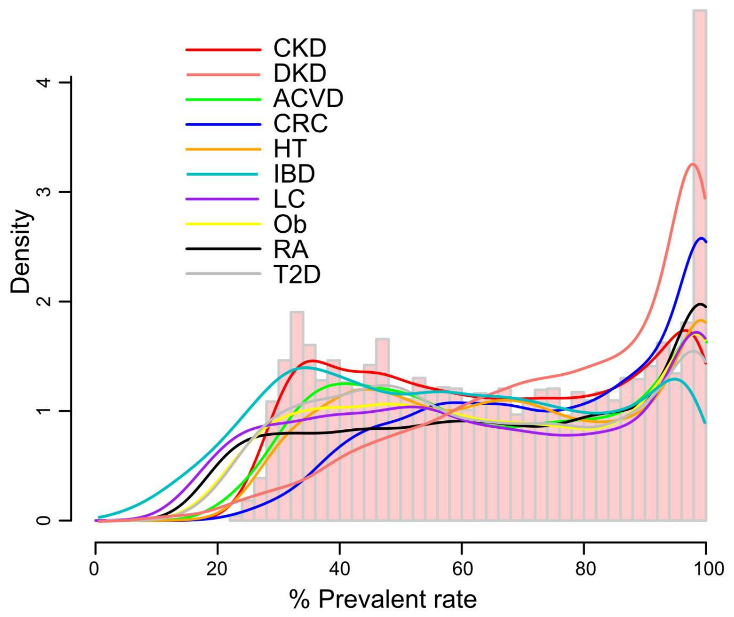

Supplementary figure 17. Detection rate of 4,649 CKD-associated vOTUs in 7 cohorts.

Barplot and the red line show the distribution of detection rate of 4,649 CKD-associated vOTUs in the current CKD study. The other colored lines show the distribution of detection rate of these vOTUs in 7 cohorts.

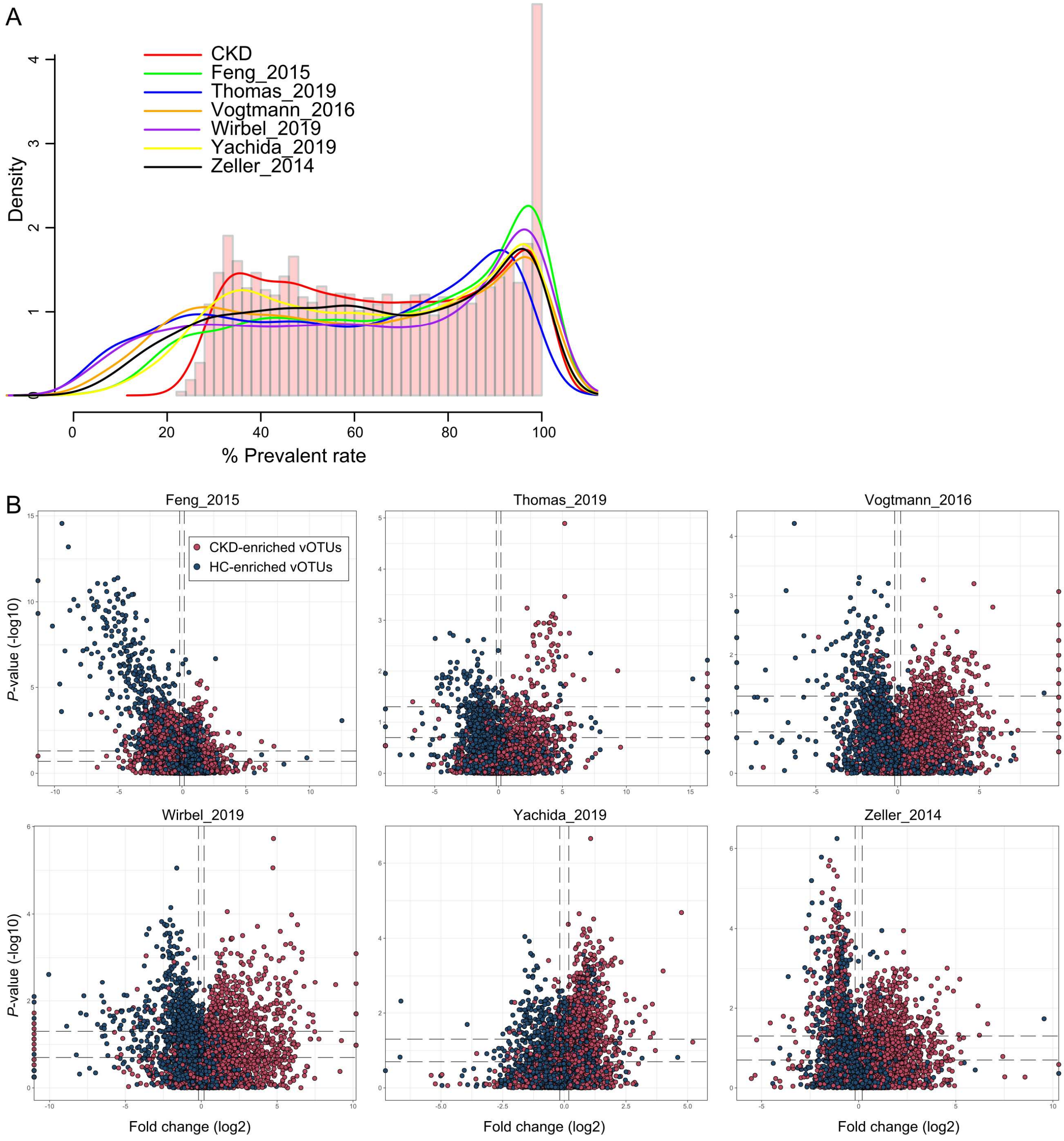

Supplementary figure 18. Alterations of CKD-associated vOTUs in 6 studies of CRC faecal microbiome.

(A) Barplot and the red line show the distribution of detection rate of 4,649 CKD-associated vOTUs in the current CKD study. The other colored lines show the distribution of detection rate of these vOTUs in 6 cohorts. (B) Volcano plots showing the fold change vs. q-values for vOTUs in 6 studies. The X-axis shows the ratio (log2 transformed) of vOTU abundance in disease cases (fold>0) compared with that in healthy controls (fold<0). The Y-axis shows the q-value (-log10 transformed) of a vOTU. vOTUs are colored by their enrichment directions in CKD patients vs. healthy controls. Horizontal dotted lines: q<0.05 and q<0.01; vertical dotted lines: fold<-1.2 and fold>1.2.
